# Automated high throughput nucleic acid purification from formalin-fixed paraffin-embedded tissue samples for next generation sequence analysis

**DOI:** 10.1371/journal.pone.0178706

**Published:** 2017-06-01

**Authors:** Simon Haile, Pawan Pandoh, Helen McDonald, Richard D. Corbett, Philip Tsao, Heather Kirk, Tina MacLeod, Martin Jones, Steve Bilobram, Denise Brooks, Duane Smailus, Christian Steidl, David W. Scott, Miruna Bala, Martin Hirst, Diane Miller, Richard A. Moore, Andrew J. Mungall, Robin J. Coope, Yussanne Ma, Yongjun Zhao, Rob A. Holt, Steven J. Jones, Marco A. Marra

**Affiliations:** 1 Genome Sciences Centre, BC Cancer Agency, Vancouver, British Columbia, Canada; 2 Centre for Lymphoid Cancers, BC Cancer Agency, Vancouver, British Columbia, Canada; 3 Department of Medical Genetics, University of British Columbia, Vancouver, British Columbia, Canada; Medical University Vienna, Center for Brain Research, AUSTRIA

## Abstract

Curation and storage of formalin-fixed, paraffin-embedded (FFPE) samples are standard procedures in hospital pathology laboratories around the world. Many thousands of such samples exist and could be used for next generation sequencing analysis. Retrospective analyses of such samples are important for identifying molecular correlates of carcinogenesis, treatment history and disease outcomes. Two major hurdles in using FFPE material for sequencing are the damaged nature of the nucleic acids and the labor-intensive nature of nucleic acid purification. These limitations and a number of other issues that span multiple steps from nucleic acid purification to library construction are addressed here. We optimized and automated a 96-well magnetic bead-based extraction protocol that can be scaled to large cohorts and is compatible with automation. Using sets of 32 and 91 individual FFPE samples respectively, we generated libraries from 100 ng of total RNA and DNA starting amounts with 95–100% success rate. The use of the resulting RNA in micro-RNA sequencing was also demonstrated. In addition to offering the potential of scalability and rapid throughput, the yield obtained with lower input requirements makes these methods applicable to clinical samples where tissue abundance is limiting.

## Introduction

Formalin Fixed Paraffin Embedded (FFPE) tissue is commonly prepared in hospital environments for pathology evaluations. The ability to profile nucleic acids purified from FFPE tissue can lead to important applications of genomic technologies to characterize archival samples and prospective cohorts where collection of fresh frozen samples is impractical.

FFPE extraction protocols often involve tedious manual manipulation and hazardous chemicals such as xylene, with the majority being exclusive to RNA or DNA [1–3]. Often, they are either column-based or involve organic phase extractions followed by alcohol precipitations, neither of which are straightforward to automate. A few commercial magnetic bead-based protocols have recently emerged, including the AxyMag FFPE (DNA/RNA/miRNA) kit (Corning), the ALINE FFPE Magapure kit (ALINE Biosciences), the Agencourt FormaPure kit (Beckman Coulter) and the VERSANT Tissue Preparation Reagents Kit (Siemens). The latter is used in a devoted automated system that can only be used for extraction and was evaluated using qRT-PCR for gene expression and mass spectrometry for single nucleotide polymorphism analyses [4,5]. The FormaPure kit was previously evaluated manually assessing RNA from FFPE samples that were ≤10 years old using qPCR [1] and microarrays [6,7].

FFPE processing and extraction result in significant fragmentation of DNA and RNA as well as sequencing artifacts resulting from chemical modification of bases [8]. In the context of Next Generation Sequencing (NGS), a stringent size selection to enrich for appropriate fragments is applied to overcome the FFPE fragmentation problem [8]. DNA damage is addressed by enzymatic treatment to repair lesions including nicks and de-aminated or oxidized bases [9–11]. Here, we demonstrate the use of a particular cocktail of such repair enzymes and the coupling of this repair reaction with regular end-polishing required as part of standard NGS library construction.

RNA sequencing requires the removal of ribosomal RNAs (rRNAs), which would otherwise dominate sequencing reads at >90% frequency [12–13]. Typically, this is achieved for eukaryotic RNAs via poly (A) capture [14] but this would result in severe 3’-end bias for FFPE as the RNA is degraded. Instead, protocols that remove rRNA via targeted enzymatic degradation or affinity chromatography are commonly applied [15–17]. Of these, an RNase H-based protocol was previously shown to be the most efficient [16–17]. However, the comparisons so far reported have included only 1–2 FFPE samples and lack information on the minimum input requirement. The robust performance of an automated version of the RNase H-based protocol with 36 samples is demonstrated here.

In this study, we have developed protocols that allow the use of up to 34 years old FFPE material for NGS of all three classes of nucleic acids (genomic DNA, RNA and microRNAs) in large scale studies. These methods involve high throughput and automated end-to-end solutions from the upstream lysis and deparaffinization steps to nucleic acid purifications and library construction.

## Materials and methods

### Samples

The samples used in this study were obtained as part of the lymphoid cancer and personalized oncogenomics projects that were approved by University of British Columbia/BC Cancer Agency Research Ethics Board (REB #H05-60103 and REB# H12-00137, respectively). Ninety one FFPE samples were chosen for the study, mainly Diffuse Large B Cell Lymphoma (DLBCL) in origin. The sample collection dates vary between 1–34 years and the size of tissue targeted was ~100 mm^2^ (including 2–5 10 μm thick scrolls).

Besides human FFPE samples, mouse FFPE samples were also used for certain experiments. The C57BL/6 mouse tissues included liver, spleen, stomach, brain, heart, kidney, and lung. The FFPE blocks from these tissues were prepared 3–4 years prior to extraction.

Universal Human Reference (UHR) total RNA was purchased from Stratagene (Catalog #740000). Tumor samples from Fresh frozen and/or fresh tissue embedded in optimal cutting temperature compound (OCT) were obtained as part of the British Columbia Cancer Agency’s personalized oncogenomics (POG) project.

### Nucleic acid extraction from FFPE tissues

An automated process for sample extraction was developed based on the FormaPure protocol and is described in detail in S1 File. Agencourt FFPE FormaPure kit (Beckman; Catalog# A33343) was used for extraction according to the manufacturer’s instructions for micro-RNA and total RNA isolation excluding DNase treatment with the following major modifications: (1) The deparaffinization/lysis step was prolonged to 2 hrs (from 1hr); and (2) a reverse cross-linking step (2 hrs incubation at 90^°^C) was included between proteinase K treatment and bead binding steps.

DNA was quantified using a Qubit dsDNA HS DNA assay (Thermo Fisher; Catalog# Q32851) or Quant-iT ™ dsDNA High-Sensitivity Assay Kit (Thermo Fisher; Catalog# Q33120). RNA was quantified using an Agilent RNA 6000 Nano Kit (Agilent Technologies; Catalog #5067–1511) or Caliper HT RNA LabChip kit (Perkin Elmer; Catalog# 760410).

Two other extraction protocols were used for comparisons. The first was the AllPrep DNA/RNA FFPE Kit (Qiagen; Catalog# 80224), performed according to the manufacturer’s instructions. The second one was a protocol developed by The Cancer Genome Atlas (TCGA) project through the Biospecimen Core Resources at Nationwide Children’s Hospital and International Genomics Consortium. The TCGA protocol involves a combination of AllPrep DNA/RNA FFPE and High Pure (Roche) kits. The protocol also includes upstream steps such as heptane-based deparaffinization that are different from those employed in either the Qiagen or Roche protocols. The DNA pellet was processed as per the aforementioned Qiagen’s protocol and the RNA supernatant was purified using the High Pure miRNA Isolation Kit (Roche; Catalog # 05 080 576 001) based on our observations that the former was superior for DNA extraction and the latter was superior for RNA extraction [Zmuda EJ *et al*, submitted].

RNA from fresh frozen/OCT-embedded tissue was extracted using the Qiagen’s AllPrep DNA/RNA Mini Kit (Qiagen; Catalog#80204) according to the manufacturer’s instructions.

### Genomic DNA libraries

The library construction protocol used for FFPE genomic DNA sequencing is described in detail in S2 File. Briefly, DNA was mechanically sheared using a Covaris’ sonicator targeting an average fragment size of 250–300 bp. FFPE lesions in DNA were repaired and simultaneously end-repair was achieved using the NEBNext FFPE End Repair reagent (New England Biolabs; Catalog# E6615B). After size selection of 200–500 bp DNA fragments using PCR Clean-DX magnetic beads (Aline Biosciences; Catalog# C-1003-450), fragments were A-tailed and adapter-ligated using the NEB Paired-End Sample Prep Premix Kit–A Tail (New England Biolabs; Catalog# E6876B) and the NEB Paired-End Sample Prep Premix Kit–Ligation (New England Biolabs; Catalog# E6877B), respectively. Following two rounds of purifications using a 1 (beads): 1(DNA) volume to volume ratio, ligated products were PCR-enriched and indexed using Phusion Hotstart (Thermo Fisher; Catalog# F540L). Amplified libraries were purified as per the post-ligation purification conditions. Libraries were quantified using a Qubit dsDNA HS DNA assay (Thermo Fisher; Catalog# Q32851) or Quant-iT ™ dsDNA High-Sensitivity Assay Kit (Thermo Fisher; Catalog# Q33120) and size profiled using an Agilent DNA 1000 Kit (Agilent Technologies; Catalog # 5067–1504) or Caliper HT DNA High Sensitivity assay (Perkin Elmer; Catalog# 760517). A pool of 32 randomly selected libraries was sequenced using an Illumina HiSeq 2500 lane (paired-end 125 bp).

### RNA libraries

The depletion of rRNAs is described in detail in S3 File. Briefly, following DNase I treatment, total RNA was used for rRNA removal using the NEBNext rRNA Depletion Kit (New England Biolabs; Catalog# E6310X). Random primed cDNA was synthesized using the Maxima H Minus 1^st^ strand cDNA synthesis kit (Thermo Fisher; Catalog# FERK1652) and the NEBNext directional second strand cDNA module (New England Biolabs; Catalog# E7750L). cDNA was mechanically sheared using Covaris’ sonicator targeting average fragment sizes of 200–300 bp. Fragments were end-repaired using a Paired-End Sample Prep Premix Kit–End Repair (New England Biolabs; Catalog# E6875B) followed by purification using 1.8 (beads): 1(DNA) volume to volume ratio. The rest of library construction steps and quantification of libraries were as described above for DNA libraries. dUTP-marked second-strand cDNA fragments were removed (thereby rendering libraries strand-specific) using the USER enzyme (New England Biolabs; Catalog# M5505L) that was included in the PCR reaction. Libraries were sequenced using Illumina HiSeq 2500 (paired-end 75 bp).

### miRNA libraries

Total RNA/nucleic acid was used for RNA ligation-based library construction as described in detail in S4 File. Briefly, 3’-end adapter was ligated to RNA using 10X T4 RNA ligase buffer (New England Biolabs; Catalog# M0242L). Following two rounds of bead-based removal of unligated adapters, 5’-end adapter was introduced using T4 RNA ligase (Ambion; Catalog# AM2141). Ligated RNA was used as a template for 1^st^ strand cDNA synthesis using Maxima H Minus reverse transcriptase (Thermo Fisher; Catalog#EP0753). Following bead-based size selection, cDNA was amplified using Phusion Hotstart (Thermo Fisher; Catalog# F540L). Amplified libraries were subsequently size-selected using a gel-based system.

### Bioinformatic analysis

Genome sequencing reads were aligned to the human reference (hg19) using BWA version 0.5.7 [18]. RNA sequencing reads were aligned to a combined RNA and genome reference (hg19 and Ensembl 61) and were subsequently processed using JAGuaR [19] to position reads spanning exon-exon junctions.

To exclude total read count as a confounding factor in quality assessment of libraries, libraries were down-sampled and duplicate marked using Picard (http://picard.sourceforge.net./). Quantification of ribosomal and mitochondrial reads was performed using BioBloom Tools [20]. Adapter-adapter dimers or adapter sequences at the ends of reads were quantified using Skewer [21]. The high coverage of the mitochondrial genome in a typical RNA-seq data together with the fact that the mitochondrial transcriptome lacks splicing, makes it a dependable reagent for the estimation of fragment size distributions. Each of the unique reads that aligned to the mitochondrial genome with the expected paired read orientation was queried for such size estimation.

## Results and discussion

**Fig 1:**
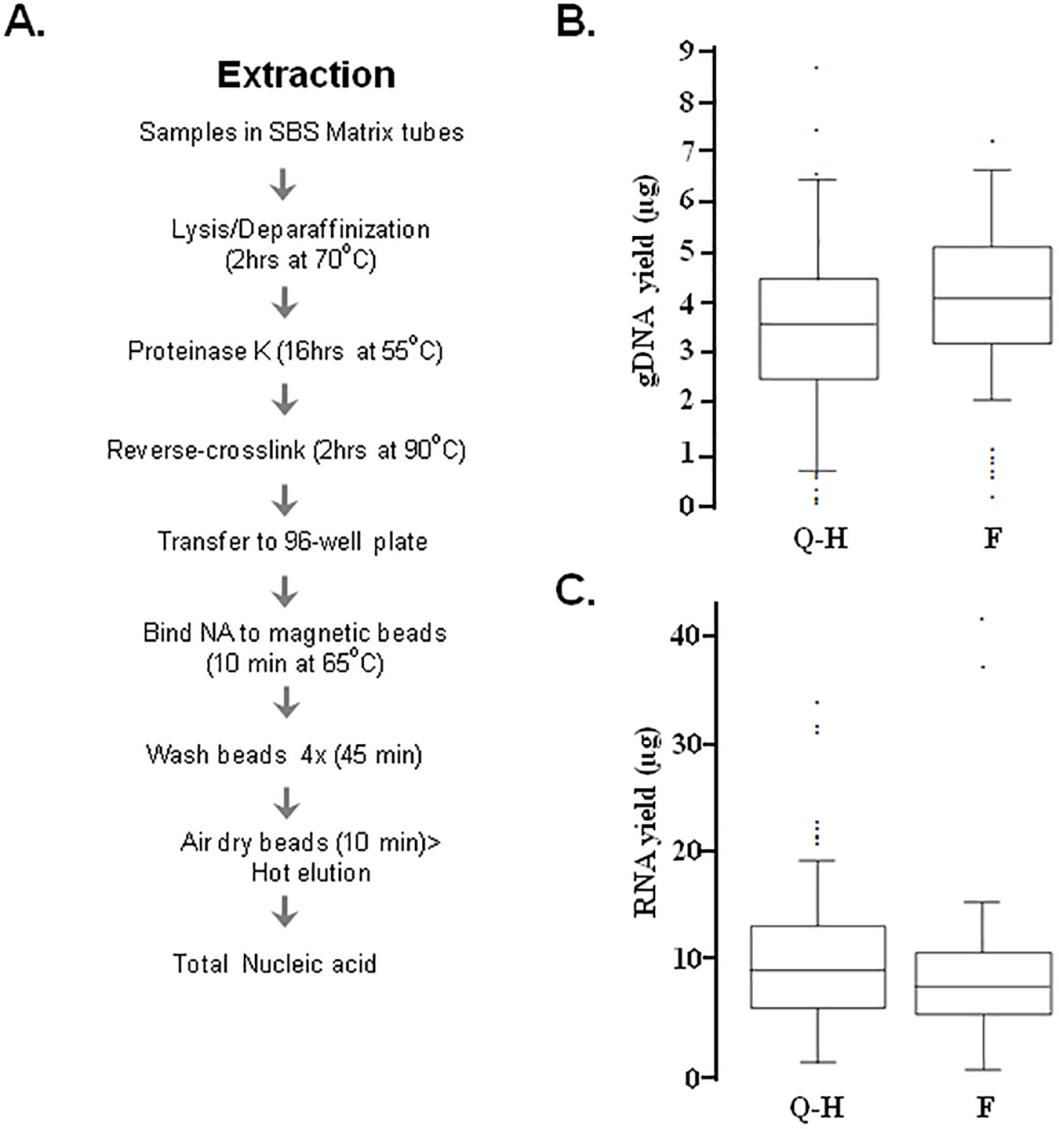
Automated high throughput FormaPure-based extraction protocol. (**A**) Work flow illustration of sample acquisition, upstream sample processing and extraction. Note that a separate high temperature incubation step is added to facilitate the reversal of remaining crosslinks. The upstream processes are manual in the original protocol whereas those steps are modified to be suitable for automation in the modified protocol. The in-house on-deck heating blocks were instrumental in rendering the lysis/deparaffinization steps automatable. Acquisition of samples in SBS format matrix tubes with their automated capping and decapping were also further measures that allowed the entire process to be amenable for automated liquid handling. (**B**) gDNA yield. Historical gDNA yield data from the Qiagen/High Pure protocol (Q; n = 142) using equivalent sizes of numerous FFPE samples of lymphoma origin was compared with that of the FormaPure protocol (F; n-91). (C) RNA yield. Comparison of the Qiagen-High Pure (Q-H), and FormaPure (F) protocols are shown. N = 142 for Q-H and N = 44 for F.

The overarching goal of the study was to develop and optimize automated laboratory methods for processing FFPE samples, with the aim of producing high quality genome and transcriptome sequencing data (Fig 1A and S1 Fig).

### High throughput and automated FFPE extraction protocol

Confirming the findings of others [22], we observed the Qiagen Allprep DNA/RNA Extraction Kit to have superior performance relative to other column-based protocols (S2 Fig). The manual lysis, deparaffinization, proteinase K treatment and reverse crosslinking of the samples as well as the automation of the subsequent purification steps on Qiagen’s QIAcube robot allows the processing of 96 samples in 8–9 separate one-day runs (with >6hrs total hands-on time). Clearly, this is still rate limiting for large scale studies and thus we sought an alternative approach that (1) could increase the throughput of extraction through automation while ensuring that nucleic acids were of sufficient quality and quantity for downstream NGS applications and (2) allowed extraction of RNA, miRNA, and gDNA in one protocol. Such alternative approach was the FormaPure kit.

We developed a version of the FormaPure protocol which included automation of lysis/deparaffinization and the addition of a reverse crosslinking step (Fig 1A). The latter resulted in up to a 3-fold increase in gDNA yield. FFPE scrolls were loaded in SBS (Society for Biomolecular Screening) format Matrix tubes, which are amenable to 96-well plate-based processing. Peltier heating blocks that were compatible with the matrix tubes and 96-well plates were designed in-house. These measures allowed automation of the extraction procedure on a Microlab Nimbus liquid handler (Hamilton Robotics).

The output from our FormaPure-based protocol is total nucleic acid. This can be used for downstream gDNA NGS application as-is. The gDNA is measured using either Qubit or Quant-iT High Sensitivity assays. Based on RNA spike-in titration of a fixed gDNA amount, we observed that these assays displayed very high specificity for DNA (S3 Fig). Extraction of 91 different FFPE samples yielded gDNA (200–7000 ng) that was sufficient for next generation sequencing analysis, at 100% sequencing success (Fig 1B). We had previously extracted DNA and RNA from 142 comparable samples from the same tissue types [23] using the column-based Qiagen/HighPure protocol developed by The Cancer Genome Atlas (TCGA) project. The DNA yield (Fig 1B) from this protocol was comparable to what we obtained using our automated FormaPure protocol.

A selection of the FormaPure extracted nucleic acid were analyzed for RNA yield after DNase treatment and bead-based purification. The FormaPure RNA was of similar quantity (Fig 1C) to that of the TCGA protocol and was within the range of what is required for a robust downstream RNA-seq protocol (100–200 ng) for 95% of the samples we evaluated. The size profile of RNA from the FormaPure shows that most of the RNA fragments were of a size suitable for RNA-seq (S4 Fig).

Of note, the Qiagen/HighPure protocol isolates the RNA and DNA sequentially and thus splitting of the lysate or the eluate is not required. This is in contrast to our FormaPure protocol wherein a portion of the eluate has to be DNase-treated for RNA preparation, thereby necessitating the proportional sacrifice of DNA yield and vice versa. Thus, the yield obtained using the FormaPure protocol is lower than what is reported in Fig 1B if both RNA and DNA are recovered. However, both protocols provide an absolute RNA and DNA yield that is far in excess of what is required for downstream NGS applications (see below).

The results described above supported the notion that the FormaPure-based protocol was robust for extraction of DNA/RNA for standard sized FFPE scrolls (~100 mm^2^). Importantly, the automated protocol allowed the processing of 96 samples in a single run of 1–2 days with <1 hr hands-on time.

### Suitability of the new FFPE extraction protocol for gDNA sequencing

**Fig 2:**
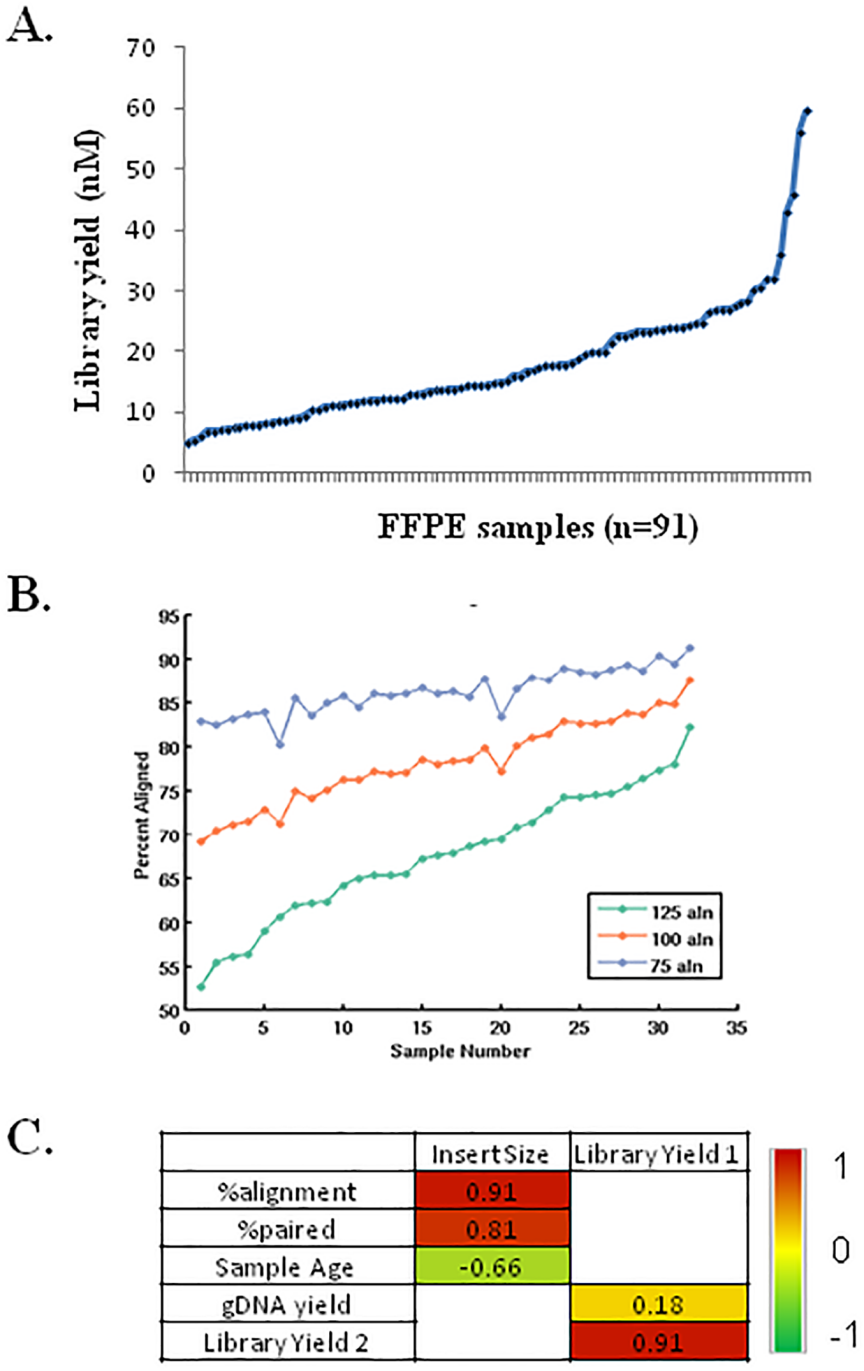
Suitability of the FormaPure extracted DNA for next generation sequencing. (**A**) Final library yield. Library yield (nM) for 100 ng of DNA extracted from each source using the FormaPure protocol (n = 91). (**B**) Alignment rate. 32 libraries that were generated using the FormaPure extraction were sequenced at PE125bp on the Illumina platform. The proportions of untrimmed reads that mapped to the human reference genome as well as alignment rates after trimming down to PE100 and PE75 are shown graphically. Data on other metrics is shown in S1 Table. (**C**) Pair-wise correlation matrix of various laboratory and bioinformatic metrics. Pearson’s correlation coefficients were calculated for the indicated pairs with the heat map representing the extent of positive (red) and negative (green) correlations.

Our new library construction protocol features an improved workflow (S1 Fig) due to (1) the replacement of post-shearing and post-PCR gel-based size selection steps with magnetic bead-based size selection steps and (2) combining into one reaction the FFPE repair enzymes with end-repair enzymes for the fragment end-polishing that is needed for standard NGS library construction, thereby eliminating a purification step. To evaluate if the FormaPure-extracted DNA provides sequencing data of acceptable quality, we constructed libraries from 91 different gDNA samples extracted from lymphoma FFPE scrolls. An input amount of 100 ng DNA was selected based on initial tests (S5 Fig) with seven cycles of PCR. All 91 libraries yielded >3 nM, which is our minimum concentration requirement for sequencing (Fig 2A) and were successfully used for Agilent SureSelect custom capture sequencing (data not shown). A pool of 32 randomly selected libraries was sequenced on a HiSeq 2500 lane. Alignment with untrimmed reads indicated an alignment rate ranging from 52–80% with the majority of libraries exhibiting 60–75% alignment rates (Fig 2B). The average insert size for these libraries was 200–250 bp (S1 Table). Thus, paired-end 125bp sequencing resulted in complete read through the sequence of interest and into the adapter sequence. Depending on the stringency required for a given aligner such as the one used here (BWA-aln), this may render a significant proportion of such reads unmappable. To directly test this, we trimmed the reads to 100 nt and 75 nt, respectively, and recalculated the alignment rate. This increased the alignment rate by ~ 10% and ~20%, respectively, (Fig 2B) suggesting that there is no significant reduction in alignment rate associated with the automated FormaPure-based extraction protocol beyond that associated with shorter fragment sizes.

One class of artifacts detected in FFPE DNA sequence analysis is chimeric reads apparently originating from segments of DNA from non-contiguous genomic loci. One of the alignment-based sequencing quality metrics we examined was percent of aligned and paired reads. Non-paired reads included chimeric reads. The average percent of paired reads for the 32 libraries was 92% versus >99% for reads from non-FFPE material (S1 Table**).** The relatively high proportion of chimeric reads in FFPE libraries might be associated with a higher proportion of single stranded fragments than in fresh frozen samples, which in turn could be due to the shorter fragmented DNA in the FFPE samples, which may denature during high temperature incubations required for FFPE DNA extraction. The T4 DNA ligase used in NGS library construction is predominantly dependent on double-stranded DNA substrates [24]. Thus, the explanation for the occurrence of the chimeric artifacts most likely lies upstream of the ligation step. We consider it possible that two single-stranded fragments, not contiguous in the genome, may anneal together due to short random complementary stretches of sequence. These short complementary regions may prime strand filling during the end-polishing part of the standard library construction process. Indeed, a previous study has demonstrated that this phenomenon is likely to occur by generating T4 DNA ligase- dependent libraries from single-stranded cDNA fragments [25]. Consistent with the notion that single stranded DNA might be accounting for the difference in percent paired reads between FFPE and fresh frozen DNA libraries that were generated using our ligation-based protocol, we observed a significant reduction in this difference when libraries were made using a double-strand DNA-specific tagmentation-based Nextera protocol was used for library construction (S6 Fig).

To assess the diversity of the ligation-based genome libraries made from nucleic acid that was extracted using our FormaPure protocol, the percent of duplicate reads was calculated and the average was found to be one percent of aligned reads (S1 Table). At comparable depth, libraries made from FFPE DNA extracted using the Qiagen protocol exhibit a similar duplicate rate but those from fresh frozen DNA tended to have duplicate rates of 0.2–0.4%. Taking these duplicate rates into account, and extrapolating from deep sequencing and subsequent down-sampling from such libraries (S7 Fig), we estimate that—>30x redundant human genome coverage can be obtained from FFPE libraries sequenced to depths of 2 to 2.5 billion reads of human genome on an Illumina’s HiSeq 2500, indicating an acceptable level of library diversity.

Reproducibility of the library construction protocol was inferred based on the high correlation of library yield obtained for two independent library preparations from all of the 91 FFPE samples (Fig 2C). Despite the input gDNA amount being normalized to 100 ng for library construction, library yield displayed positive, albeit weak, correlation with gDNA yield following extraction (Fig 2C). There was a strong positive correlation of the computationally calculated insert size with percent alignment and percent paired metrics, respectively (Fig 2C).

Taken together, the data discussed above indicate that our automated FormaPure extraction protocol yields DNA that is suitable for genome sequencing.

### Suitability of the new FFPE extraction protocol for total RNA-seq

**Fig 3:**
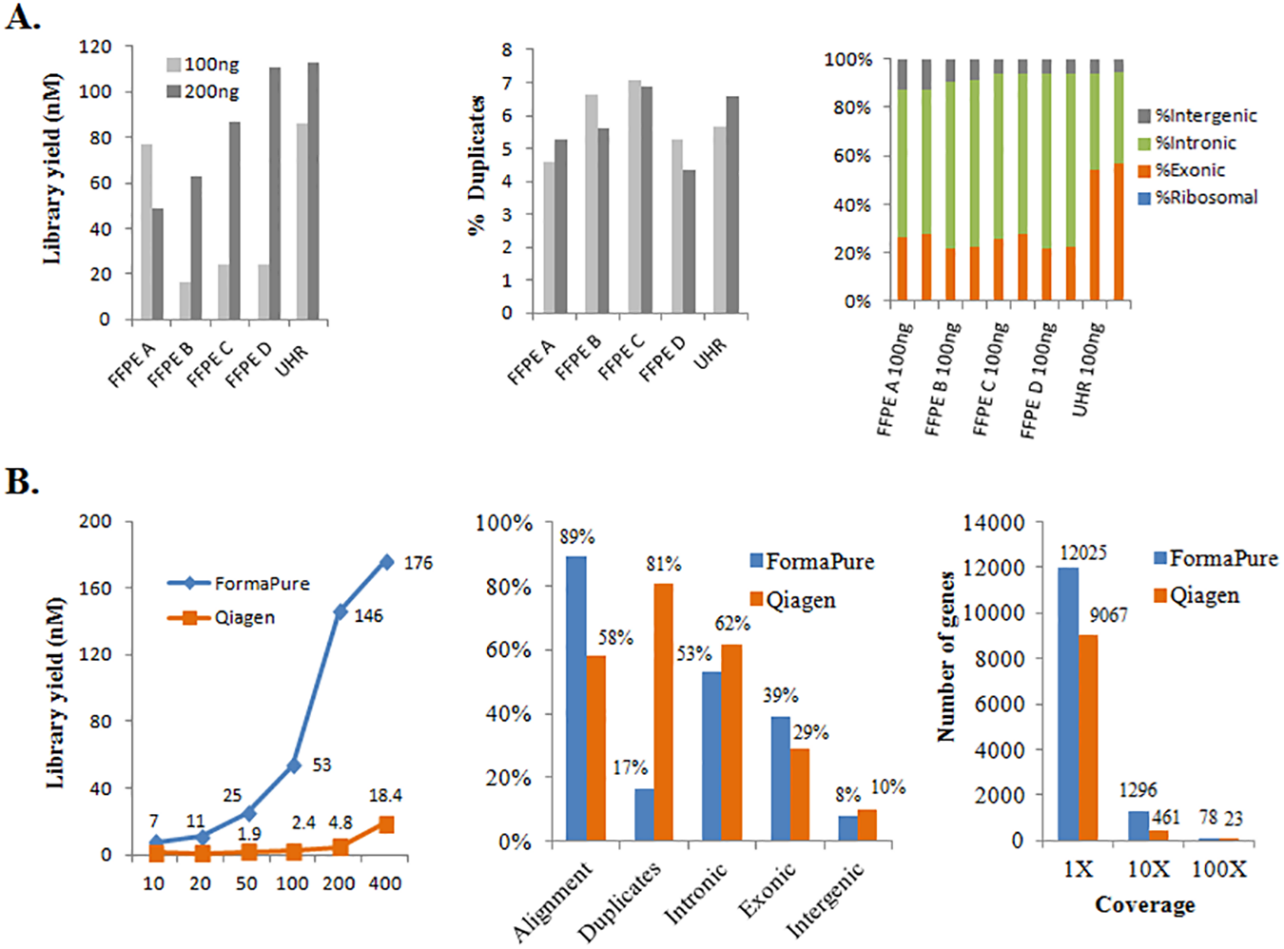
Suitability of the FormaPure extracted RNA for FFPE strand-specific RNA-seq. (**A)** Strand-specific libraries were generated from four different FormaPure extracted human FFPE samples (FFPE A-D) and UHR fresh RNA. Two different total (DNase-treated) RNA input amounts were used (100 and 200 ng, respectively). Final library yield (nM) (left panel) and % duplicates (middle panel) as well as the distribution of aligned reads to various regions of the transcriptome (right panel) are shown graphically. These libraries were sequenced as a pool at PE75 bp. (**B**) Comparison of Qiagen and FormaPure extraction protocols using mouse FFPE scrolls. Final library yield (nM) (Left panel) and % duplicates, % aligned, and the distribution of aligned reads to various regions of the transcriptome (middle panel) as well as number of genes with 1x coverage (right panel) are shown graphically.

We have used the NEBNext® rRNA Depletion Kit (Human/Mouse/Rat) for high quality library construction from fresh RNA samples, using as little as as 20 ng of total RNA (S8 Fig). Here, we assess the performance of this protocol for FFPE RNA. For this purpose, we used 100 ng and 200 ng of DNase-treated RNA obtained using our modified FormaPure-based protocol on four lymphoma samples. 300 ng or 400 ng input amounts were also used for three of the samples. Following rRNA depletion, strand-specific libraries were generated using our newly optimized protocol [Haile et al, in revision] with 13 cycles of PCR. Libraries were also made from corresponding amounts of high quality, Universal Human RNA (UHR) to serve as references. Libraries yielded 5–40 times our minimum requirement of 3nM (Fig 3A, left panel). The 200 ng input libraries gave higher yield than the 100 ng libraries for three out of four of the FFPE samples. Library quality data following sequencing is presented in S2 Table. All FFPE libraries have high alignment rates (90–95%) that were only marginally lower than those of UHR libraries (S2 Table). rRNA content was low at <0.01% for all the FFPE libraries as well as the UHR control (Fig 3A, right panel; S2 Table). The duplicate rate was comparable for all libraries for input amounts ranging from 100 to 400 ng and was only slightly higher than duplicate rates from corresponding UHR libraries (Fig 3A, middle panel; S2 Table).

Analysis of reads that mapped to intronic, exonic and intergenic regions indicated that the FFPE libraries had much lower exon content as compared to the UHR libraries (Fig 3A, right panel). This is consistent with other reports [16–17] that compared libraries from fresh frozen and FFPE samples, all of which employed column-based extraction protocols and either RNase H-based or RiboZero protocol for rRNA depletion. The consistently lower exon content across diverse protocols suggests a feature(s) related to FFPE RNA is likely to be the explanation. We consider it possible that nuclear pre-mRNA and processing intermediates that harbor intron sequences are preferentially protected from degradation, compared to cytosolic mature RNAs that may be more labile. Indirect support for this hypothesis is the observation that exon content may vary depending on the extraction method applied, which ultimately relates to the extent of disruption of the nucleus [26]. This dependency on extraction method is more severe for rRNA depletion protocols than poly (A)-capture protocols, which may be associated with the observation that nuclear RNAs including pre-mRNAs are not significantly polyadenylated [26].

To further validate our FFPE RNA-seq protocol beyond the four lymphoma samples, we made libraries from 36 additional FFPE samples. Of these, five were extracted using of the Qiagen protocol and the rest were extracted using the modified FormaPure protocol. Three fresh frozen samples and UHR were also included in this experiment. Even though we aimed for 100 ng RNA input, we recovered variable RNA amounts after DNase treatment and bead-based purification. In total, five libraries were prepared using 30–70 ng RNA, 11 were prepared using 70–93 ng and the remainder were from 100 ng DNase-treated RNA input. All but one library passed the minimum library concentration required (S9A Fig). qRT-PCR was used to assess the ribosomal RNA content of these libraries (S9B Fig). Based on historical data, where we had both qRT-PCR and RNA-seq data representing various rRNA content for the same libraries (S10 Fig), we anticipate that the ribosomal content would fall below 2% of reads for the majority of these 36 FFPE libraries if they were to be sequenced.

We next evaluated the technical reproducibility of our FFPE RNA-seq protocol. We made two different libraries from the same sample that involved independent DNase treatment, rRNA depletion, library construction and sequencing. This process was then repeated for another sample. We then considered genes with non-zero read counts, performing Pearsoncorrelation analysis between the two pairs of libraries, with correlation coefficients of 0.98–0.99 (S11 Fig).

To provide a direct comparison of RNA sequencing quality for the same sample extracted using the standard Qiagen protocol and our automated FormaPure protocol, we used mouse FFPE scrolls from various tissues. Total RNA/total nucleic acid was extracted and pooled for each of the extraction protocols respectively. For the FormaPure extracted pool of total nucleic acid, RNA was measured directly without DNase treatment. “RNA” was titrated from 400 ng down to 10 ng and was treated with DNase I before rRNA depletion followed by library construction. Library yield from the FormaPure extracted RNA was 7 to 30-fold higher than that of Qiagen extracted RNA **(**Fig 3B, left panel). This is unlikely to be due to gDNA contamination as sequence analysis of the 100 ng libraries revealed that the intronic and intergenic content were lower in the FormaPure libraries **(**Fig 3B, middle panel). This is consistent with the fact that samples were all treated with the maximum amount of DNase I and residual gDNA was further removed by a second DNase I treatment that was part of the RNase H-based rRNA depletion protocol. The second DNase I treatment, which is part of the rRNA depletion kit, was required for removal of rRNA DNA probes. The higher quality of the FormaPure libraries was also reflected in the higher alignment rates, lower duplicate rates and higher numbers of genes detected **(**Fig 3B, middle and right panels). Of note, the exon content for the FormaPure libraries (despite being lower than that observed for fresh frozen) was higher than that of the Qiagen extracted FFPE libraries **(**Fig 3B, middle panel).

Taken together, these data validate our FFPE RNA-seq protocol as robust for input material as low as 100 ng total RNA and also demonstrates that the quality of RNA extracted using our automated FFPE-based protocol is more suitable for RNA-seq than FFPE RNA extracted using the Qiagen protocol.

### Suitability of the new FFPE extraction protocol for microRNA sequencing

**Fig 4:**
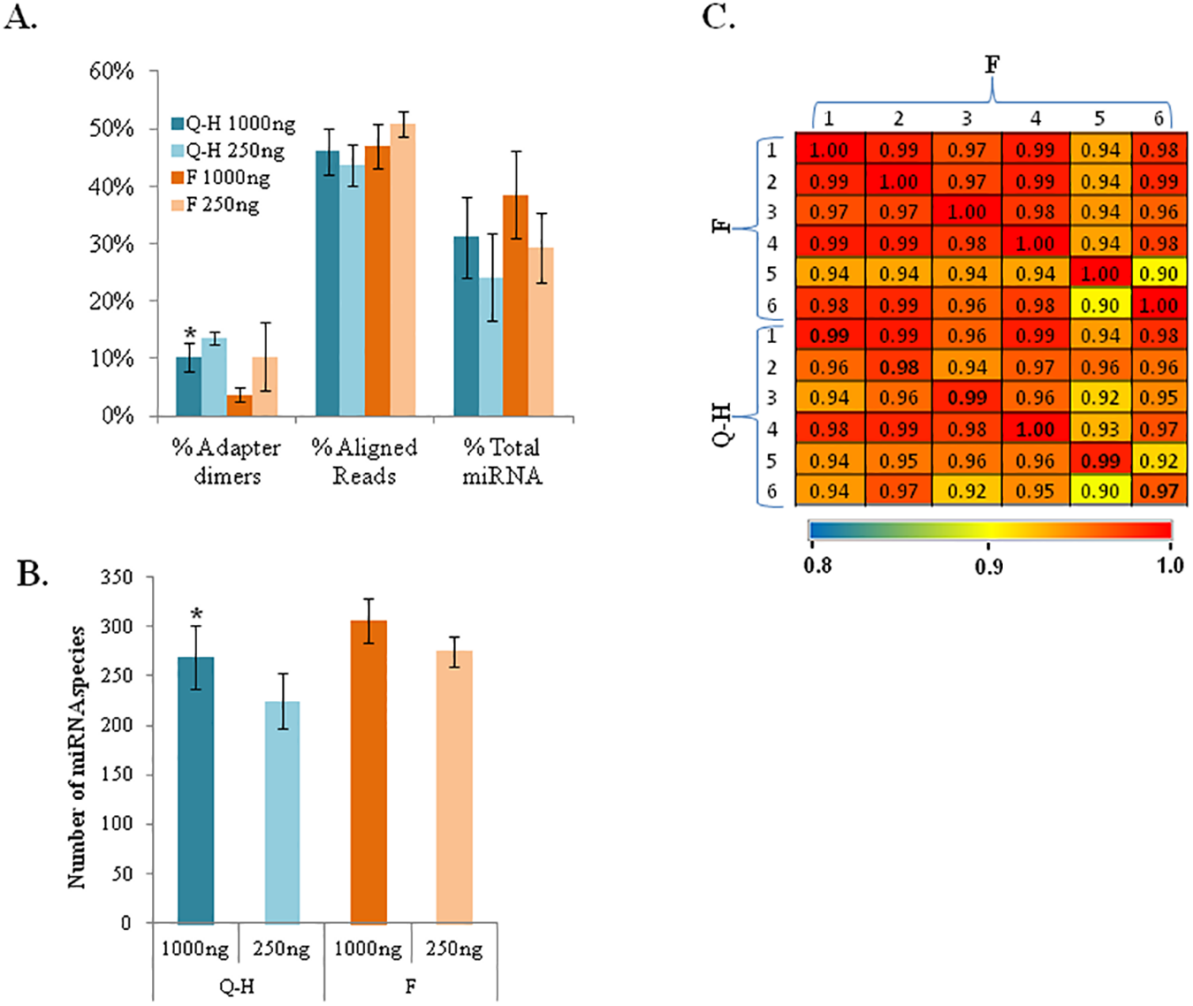
Suitability of the FormaPure extracted RNA for FFPE miRNA-seq. (**A**) miRNA library quality. Comparisons of adapter dimer content, alignment rate, and miRNA content between the FormaPure (F) protocol and the Qiagen-High Pure (Q-H) protocol for 250 ng and 1000 ng input amounts are shown. n = 6 for the 1000 ng F and Q-H conditions; n = 4 for the 250 ng F condition and n = 3 for the 250 ng Q-H condition. *P<0.05. Error bars = standard deviations. **(B)** miRNA library diversity. Comparison of the number of miRNA species with ≥10 reads between the two protocols. (**C**) Inter-protocol expression correlation. The strength of the expression correlation of miRNAs from samples that were extracted using the FormaPure protocol with that of matching samples from the Qiagen-High Pure protocol is shown in the heat map. In bold are correlation values between the two protocols from matched samples.

The version of the FormaPure protocol we modified is claimed by the manufacturer to retain small RNAs. To test this assertion, we compared miRNA libraries that we generated from RNA extracted using this extraction protocol with those that were previously generated from matching samples that were extracted using the TCGA protocol (n = 14). Overall, the quality of the libraries was slightly higher for the FormaPure protocol. For example, adapter dimer content was 10% in the TCGA protocol versus 4% in the FormaPure protocol (Fig 4A). miRNA diversity was greater in the FormaPure protocol with 269 miRNA species with ≥ 10 reads in the TCGA protocol versus 306 miRNA species in the FormaPure protocol for the 1000 ng input (Fig 4B). Despite these differences, correlation of miRNA expression in matched samples extracted using both protocols was relatively high (Pearson’s correlation = 0.97 to 1.00) (Fig 4C). Importantly, the inter-protocol similarities for matched samples tended to be higher than what was observed among different samples within either protocol. Of note, the low proportion of miRNA reads in both extraction protocols (Fig 4A) is likely due to degradation of miRNAs in FFPE RNA as reported previously [27]. Taken together, these data indicate that the FormaPure protocol yields adequate miRNA for library sequencing of reasonable diversity and quality.

In conclusion, this study has established an automated suite of protocols that allow for large-scale transcriptome and genome studies of archived samples. The integrated automated workflow spans all steps, allowing high throughput removal of paraffin, reversal of formaldehyde crosslinks, extraction of RNA and gDNA, and library construction for Illumina sequencing. This set of protocols is developed while ensuring data quality is maintained, and most importantly, meeting input requirements for clinical samples.

## Supporting information

S1 Fig Library construction workflows. Left panel: genome; middle panel: RNA-seq; right panel: miRNA.
(TIF)

S2 Fig Comparison of extraction protocols. RNA yield and size profile is using Agilent RNA Nano assay.
(TIF)

S3 Fig The effect of RNA in gDNA quantification. gDNA was spike-in with various RNA concentrations and the mixture was measured by Qubit DNA high sensitivity assay.
(TIF)

S4 Fig The quality of RNA extracted using the modified FormaPure protocol. RNA was assayed on Agilent RNA Nano.
(TIF)

S5 Fig Input titration. Input amounts are as indicated from three different FFPE sources. % aligned (left panel) and % duplicates (right panel) upon sequencing of the resulting libraries are shown.
(TIF)

S6 Fig Differences in % paired reads between ligation-based and tagmentation-based (Nextera) library construction protocols. Starting gDNA amount was 20 ng from FFPE material.
(TIF)

S7 Fig Coverage versus duplicate estimate at lower depth. The coverage values (Y-axis) were calculated based on 2–2.5 billion reads whereas the duplicate percentages (X-axis) were calculated after down sampling of the same data to 10 million reads.
(TIF)

S8 Fig Input titration and total RNA-seq libraries from fresh RNA. Library yield is shown in (A). duplicate rate of resulting libraries is shown in (B). Besides UHR RNA, RNA from OCT-embeded tumor samples (POGs) were also assessed.
(TIF)

S9 Fig Larger scale evaluation of FormaPure extracted RNA in RNA-seq library construction. Library yield is shown in (A). qRT-PCR measurement of 18s rRNA relative to GAPDH is shown in (B).
(TIF)

S10 Fig Estimation of rRNA content via qRT-PCR versus RNA-seq. qRT-PCR values (Y-axis) were derived from the ratio of 18s rRNA to GAPDH levels. X-axis represents % rRNA as calculated upon sequencing of the same RNA libraries.
(TIF)

S11 Fig Technical reproducibility of FFPE RNA-seq. Log-log plot of expression correlation between independently generated pairs of libraries from the same sample (for two different sources) are shown.
(TIF)

S1 File Extraction protocol. (PDF)

S2 File Genome library construction protocol. (PDF)

S3 File rRNA depletion protocol. (PDF)

S4 File miRNA library construction protocol. (PDF)

S1 Table Sequencing quality metrics for genome libraries. (XLS)

S2 Table Sequencing quality metrics for RNA-seq libraries. (XLS)
