# Supplementary figures and images for: Automated high throughput nucleic acid purification from formalin-fixed paraffin-embedded tissue samples for next generation sequence analysis
Source: PLoS One. 2017 Jun 1;12(6):e0178706. doi: 10.1371/journal.pone.0178706 (PMC5453589; doi:10.1371/journal.pone.0178706)

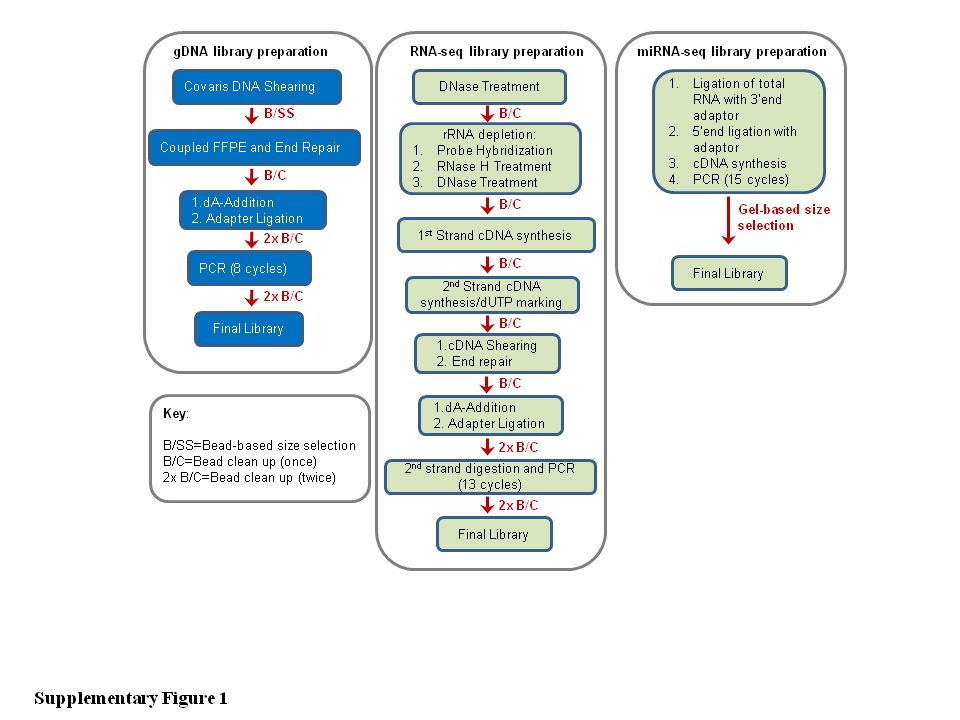

Supplement: S1 Fig — Left panel: genome; middle panel: RNA-seq; right panel: miRNA. (TIF) [file pone.0178706.s001.TIF]

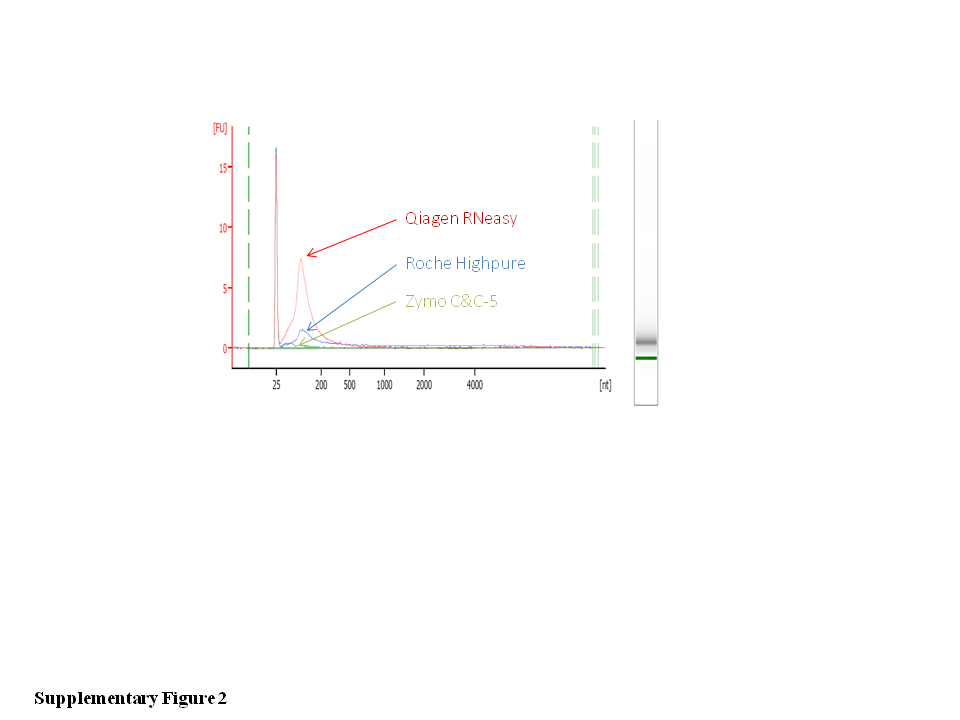

Supplement: S2 Fig — RNA yield and size profile is using Agilent RNA Nano assay. (TIF) [file pone.0178706.s002.TIF]

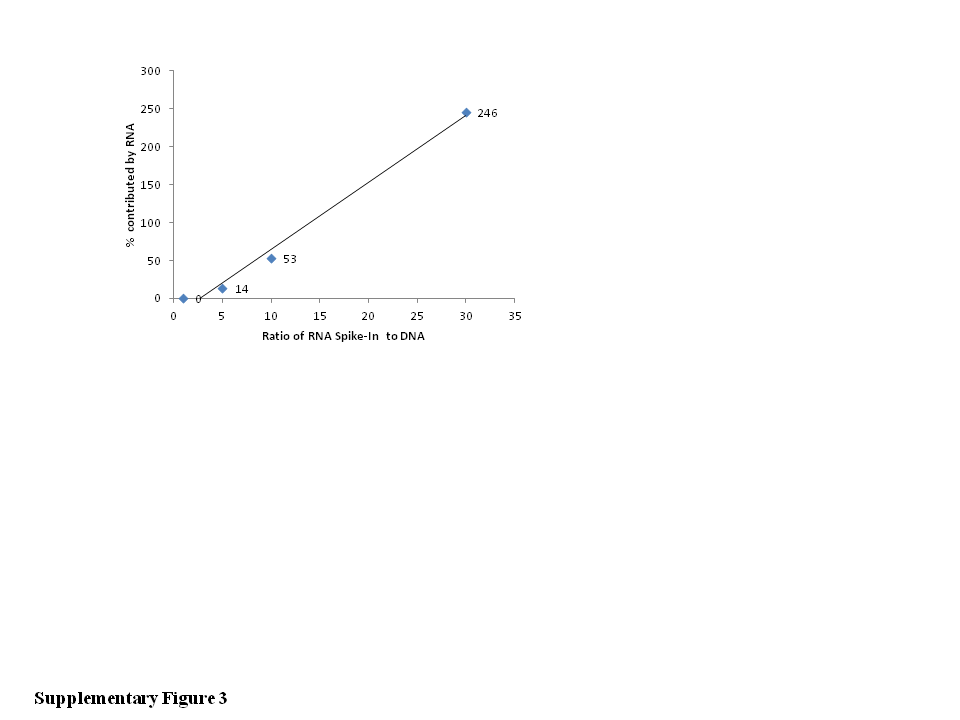

Supplement: S3 Fig — gDNA was spike-in with various RNA concentrations and the mixture was measured by Qubit DNA high sensitivity assay. (TIF) [file pone.0178706.s003.TIF]

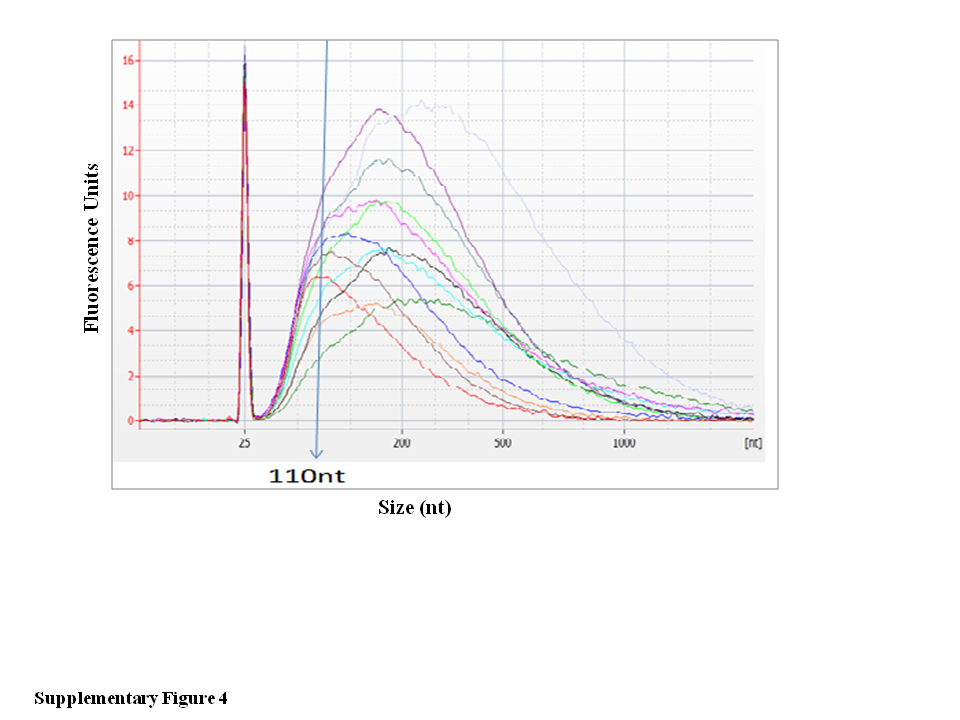

Supplement: S4 Fig — RNA was assayed on Agilent RNA Nano. (TIF) [file pone.0178706.s004.TIF]

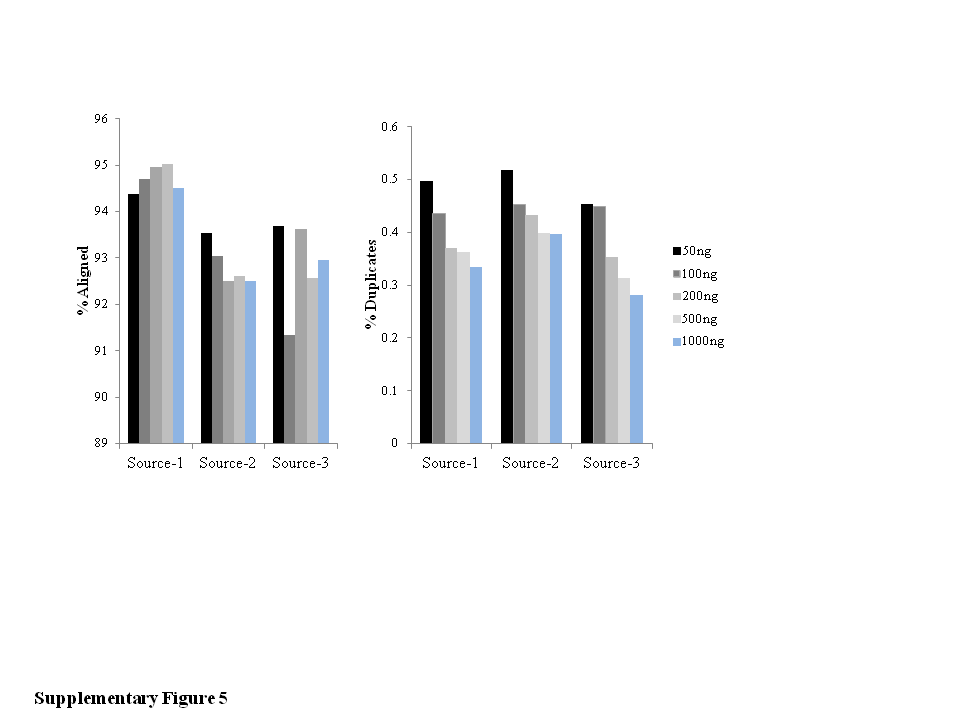

Supplement: S5 Fig — Input amounts are as indicated from three different FFPE sources. % aligned (left panel) and % duplicates (right panel) upon sequencing of the resulting libraries are shown. (TIF) [file pone.0178706.s005.TIF]

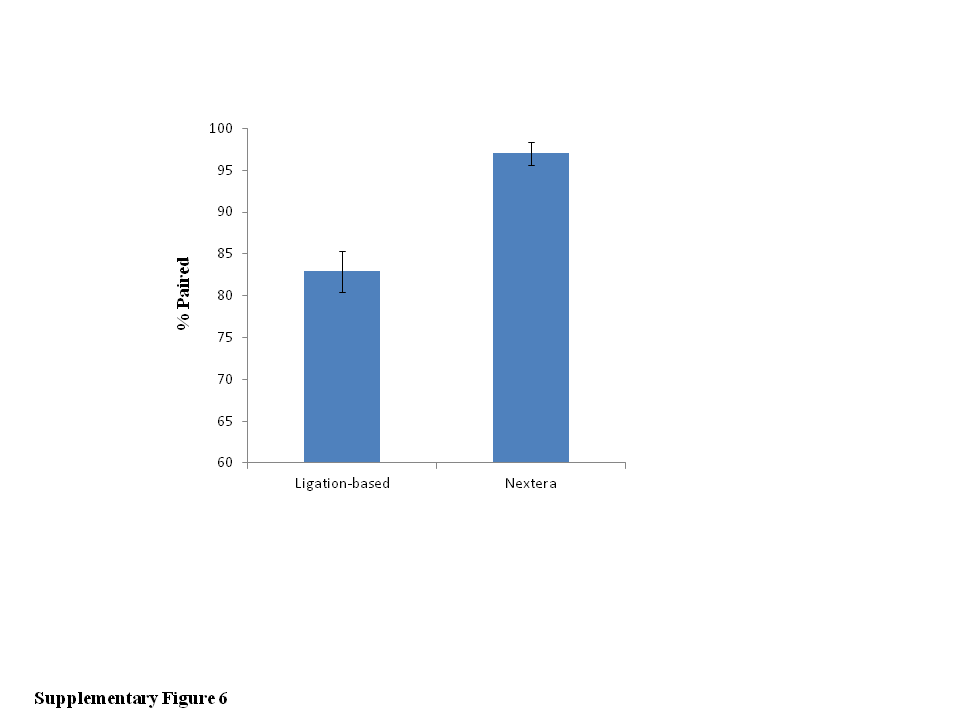

Supplement: S6 Fig — Starting gDNA amount was 20 ng from FFPE material. (TIF) [file pone.0178706.s006.TIF]

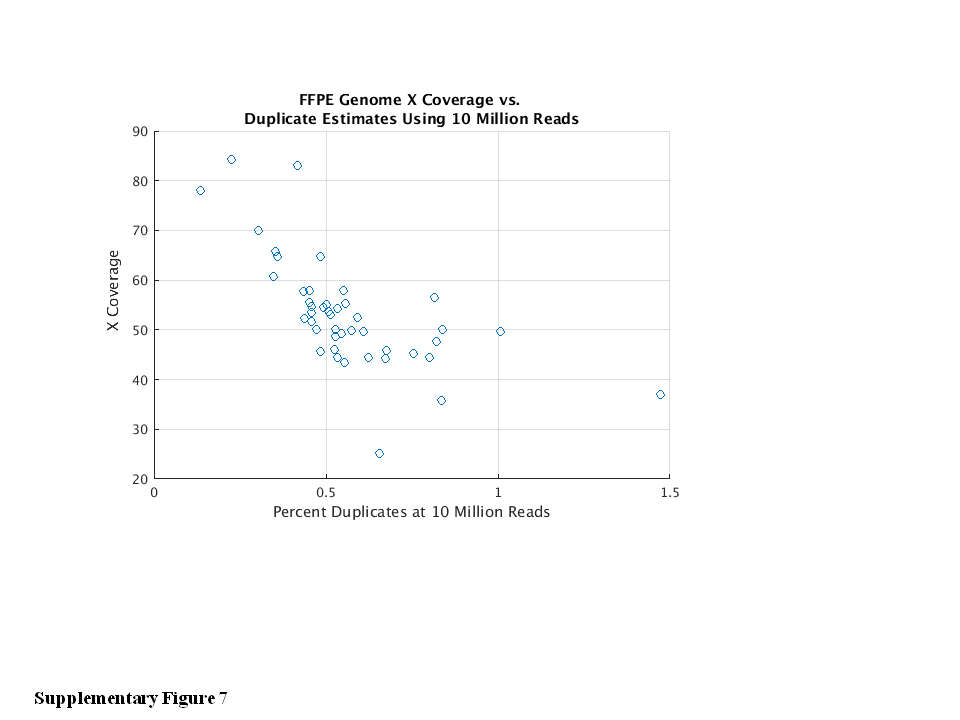

Supplement: S7 Fig — The coverage values (Y-axis) were calculated based on 2–2.5 billion reads whereas the duplicate percentages (X-axis) were calculated after down sampling of the same data to 10 million reads. (TIF) [file pone.0178706.s007.TIF]

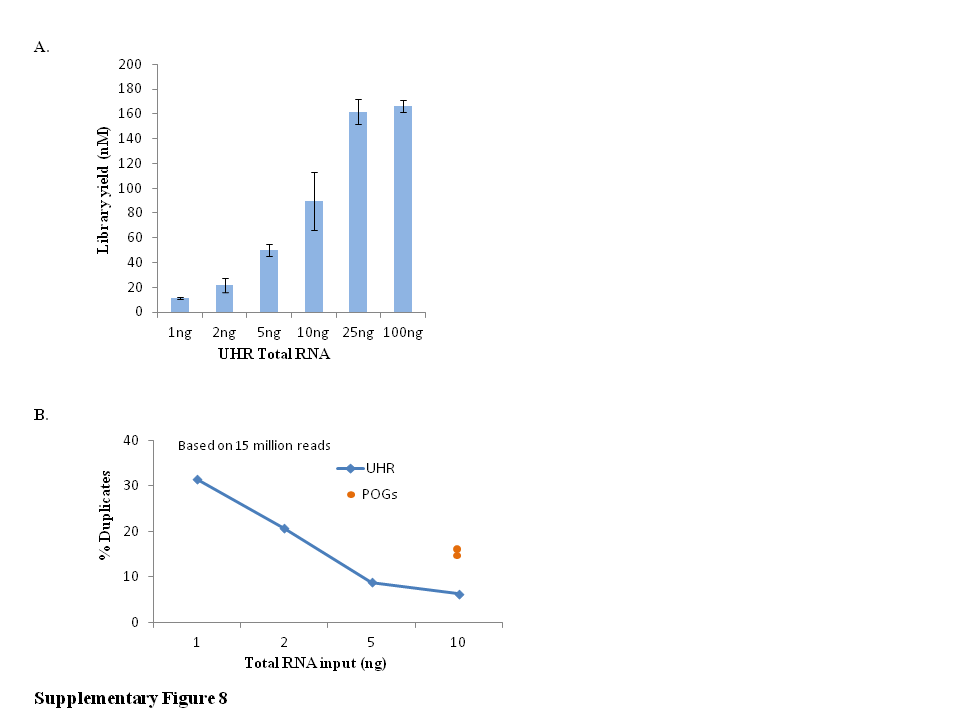

Supplement: S8 Fig — Library yield is shown in (A). duplicate rate of resulting libraries is shown in (B). Besides UHR RNA, RNA from OCT-embeded tumor samples (POGs) were also assessed. (TIF) [file pone.0178706.s008.TIF]

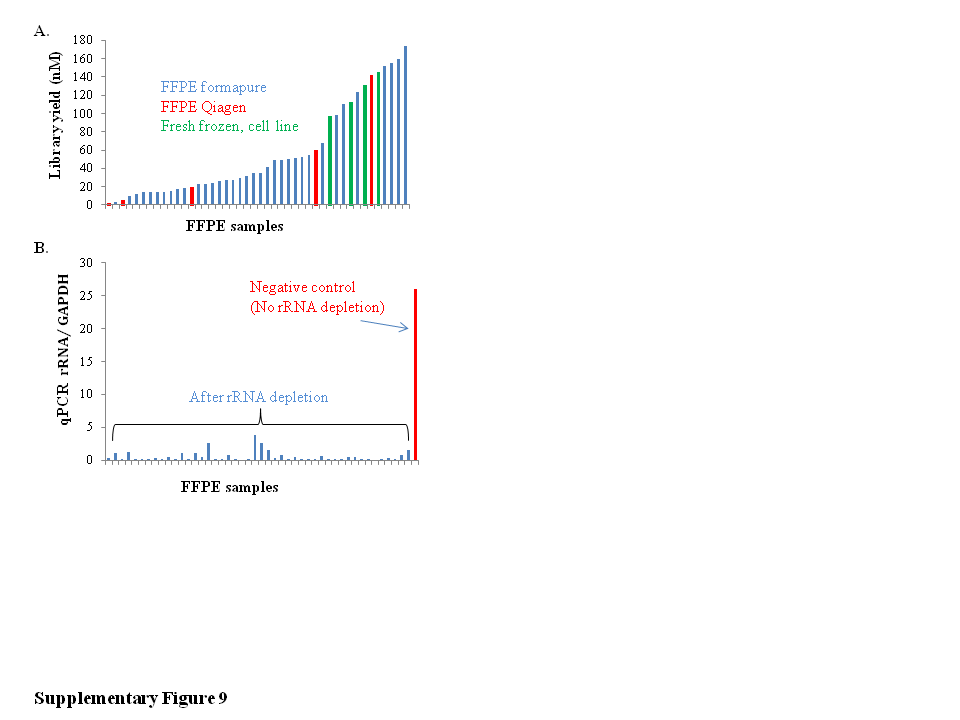

Supplement: S9 Fig — Library yield is shown in (A). qRT-PCR measurement of 18s rRNA relative to GAPDH is shown in (B). (TIF) [file pone.0178706.s009.TIF]

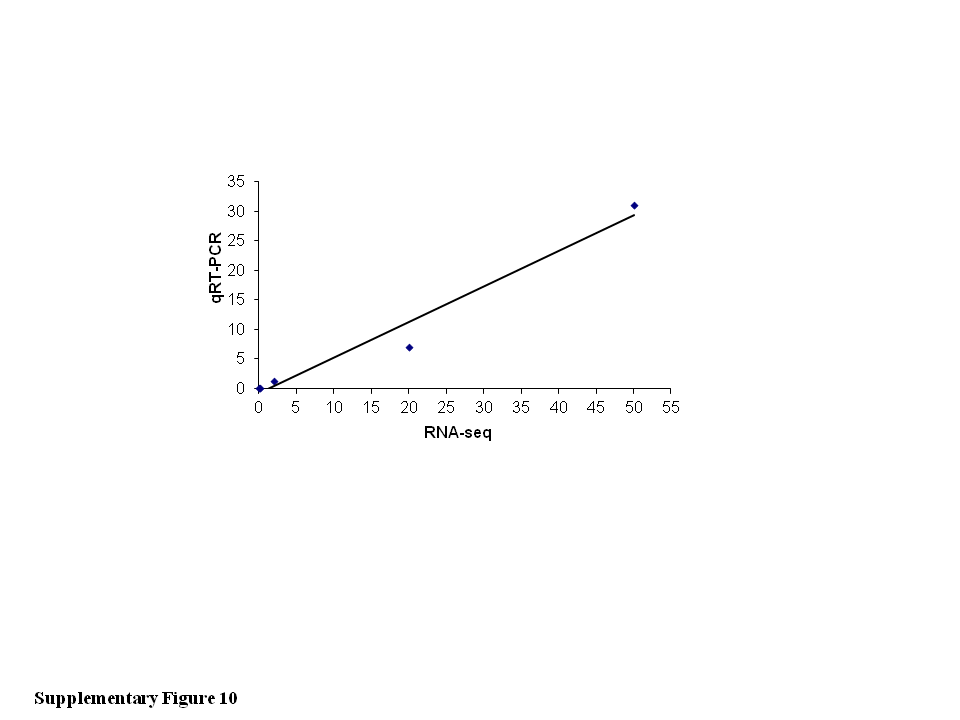

Supplement: S10 Fig — qRT-PCR values (Y-axis) were derived from the ratio of 18s rRNA to GAPDH levels. X-axis represents % rRNA as calculated upon sequencing of the same RNA libraries. (TIF) [file pone.0178706.s010.TIF]

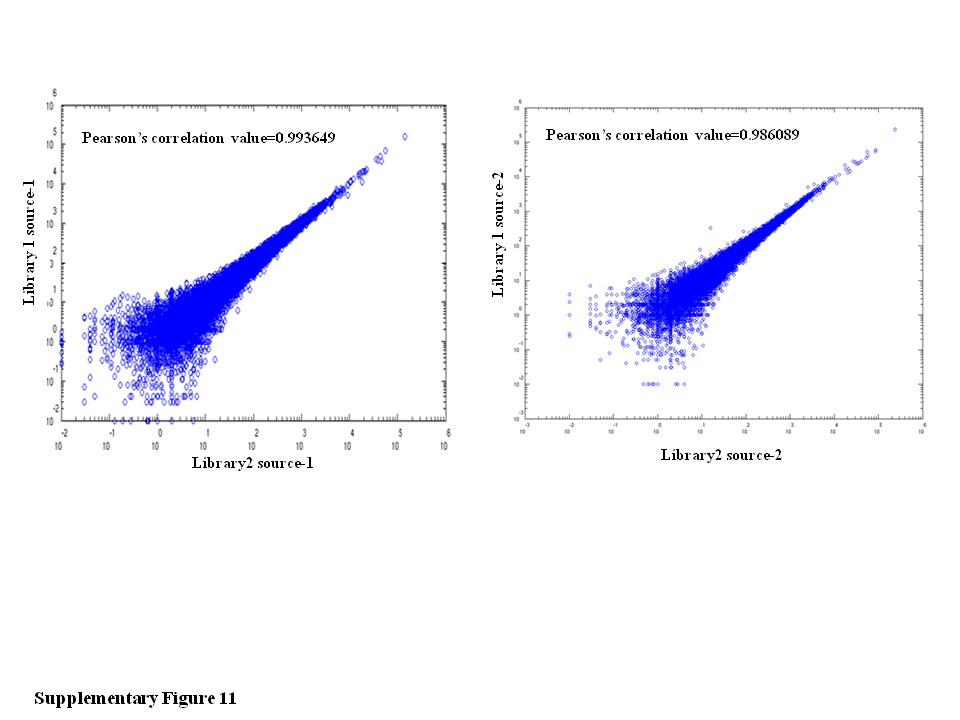

Supplement: S11 Fig — Log-log plot of expression correlation between independently generated pairs of libraries from the same sample (for two different sources) are shown. (TIF) [file pone.0178706.s011.TIF]
