# Supplementary material for: Automated high throughput nucleic acid purification from formalin-fixed paraffin-embedded tissue samples for next generation sequence analysis
Source: PLoS One. 2017 Jun 1;12(6):e0178706. doi: 10.1371/journal.pone.0178706 (PMC5453589; doi:10.1371/journal.pone.0178706)
Supplement: S1 File — (PDF) [file pone.0178706.s012.pdf]

Non Controlled Version
**Note: Controlled Versions of this document are subjected to change without notice*

Total Nucleic Acid Extraction From Formalin Fixed Paraffin Embedded Tissues on the Nimbus

I. Purpose

To isolate and purify total Nucleic Acids from formalin fixed paraffin embedded (FFPE) tissues for Next Generation Sequencing.

II. Scope

All procedures are applicable to the BCGSC Library Construction and TechD Groups.

III. Policy

This procedure will be controlled under the policies of the Genome Sciences Centre, as outlined in the Genome Sciences Centre High Throughput Production Quality Manual (QM.0001). Do not copy or alter this document. To obtain a copy see a QA associate.

IV. Responsibility

It is the responsibility of all personnel performing this procedure to follow the current protocol. It is the responsibility of the Library Construction Group Leader to ensure personnel are trained in all aspects of this protocol. It is the responsibility of Quality Systems Team to audit this procedure for compliance and maintain control of this procedure.

V. References

Document Title	Document Number
AGENCOURT® FORMAPURE® KIT NUCLEIC ACID ISOLATION FROM FORMALIN-FIXED, PARAFFINEMBEDDED TISSUE	Part# A33343

VI. Related Documents

Document Title	Document Number
Decapping and Capping Tubes using Capit-All	BSCore_WorkInst.0018
96-well DNA Quantification using the dsDNA Quant-iT High Sensitivity Assay Kit and VICTOR^{3}V	BSCore.0024

DRAFT

Non Controlled Version
**Note: Controlled Versions of this document are subjected to change without notice*

Document Title	Document Number
Checking DNA Quality using the E-Gel 48-lane E-Base Integrated Power System	BSCore.0011

VII. Safety

All Laboratory Safety procedures will be complied with during this procedure. The required personal protective equipment includes a laboratory coat and gloves. See the material safety data sheet (MSDS) for additional information.

VIII. Materials and Equipment

Name	Supplier	Number	Model or Catalogue #	
8-Channel Handheld Screw Cap Capper/Decapper for Matrix-style tubes	Thermo Scientific	4105MAT		✓
1.2mL Low Profile Plate, AB1127	Abgene	AB-1127	✓	
1000µl Rainin tips	Rainin	RT-L1000F	✓	
20µl Pipet-Lite	Rainin	L12-20	✓	
2.0 mL 96 Deep Well Waste Plate	Axygen	P-2ML-SQ-C-S	✓	
20µl Rainin tips	Rainin	RT-L10F	✓	
200µl Pipet-Lite	Rainin	L12-200	✓	
200µl Rainin tips	Rainin	RT-L200F	✓	
Agencourt, FFPE FormaPure – Large	Beckman	A33343		✓
Anhydrous Ethyl Alcohol (100% Ethanol)	Commercial Alcohol	People Soft ID:23878		✓
AB1000 96-well 200ul PCR plate	Fisher	AB1000		✓
Bench Coat (Bench Protection Paper)	Fisher	12-007-186		✓
Black ink permanent marker pen	VWR	52877-310		✓
DEPC water	Ambion	9922		✓
Disposable Trough	VWR	21007-972		✓
Distriman Repeater	Mandel	GF-3005		✓
Falcon pipettor, equ2420 PAF-2	VWR	357590	✓	
Fisherbrand Textured Nitrile gloves - large	Fisher	270-058-53		✓
Gilson distriman, Distri-2	Mandel	GF-F164001	✓	
Gilson P10 pipetman	Mandel	GF-44802		✓
Gilson P1000 pipetman	Mandel	GF-23602		✓
Gilson P2 pipetman	Mandel	GF-44801		✓
Gilson P20 pipetman	Mandel	GF23600		✓
Gilson P200 pipetman	Mandel	GF-23601		✓
Hamilton Microlab Nimbus96	Hamilton		✓	
Ice bucket – Green	Fisher	11-676-36		✓
IPA (2-Propanol) 4L	Fisher	A464-4		✓
Large Kimwipes	Fisher	06-666-117		✓
Large Volume Magnet Plate	Alpaqua	96M-EX	✓	

DRAFT

Mandel P1000 DF1000 tips	Mandel	GF-F171703		✓
Mandel P200 DF200 tips	Mandel	GF-F171503		✓
MicroAmp Clear Adhesive Film	ABI	4306311	✓	
Mini-centrifuge	Eppendorf	5417R		✓
MultiMACS Separation Unit, equ2250	Miltenyi Biotec	120-004-352		✓
Gel Imager	In House		✓	
Nimbus 1000 uL CO-RE filter tips	Hamilton	235821		✓
Nimbus 300 uL CO-RE filter tips	Hamilton	235903		✓
P200 Multichannel, 20-200uL, equ1325	Ranin	L-20-12	✓	
Peltier Heaters	In-house	NA		✓
Plate, 96-Well reservoirs, diamond-bottom, Low-Profile	Ultident	24-RES-SW96-LP		✓
Plate, 384-Well reservoirs, diamond-bottom, Low-Profile	Ultident	24-RES-SW384-LP		✓
Qiagen Elution Buffer	Qiagen	19086	✓	✓
Rainin AutoRep E	Mettler Toledo Inc	AR-E1		✓
Rainin Encode Tip, sterile, 12.5mL	Mettler Toledo Inc	ENL-12MLS		✓
Reservoir 12-Channel	Reservoir 12-Channel	RES-MW12LPSI	✓	
RNAse free 1.5 ml eppendorf tube	Ambion	12400		✓
RNAse Zap	Ambion	9780		✓
Small Autoclave waste bags 10"X15"	Fisher	01-826-4		✓
VX-100 Vortex Mixer	Rose Scientific	S-0100		✓
Wet ice	In house	N/A	N/A	N/A
Reservoir, 96-well, high profile	E&K Scientific	EK-2035		✓
Autoclave waste bags 12" x 24"	Fisher	01-826-5		✓

IX. Procedure

1. Reagent Preparation Guidelines

1.1. General Remarks on Handling RNA:

1.2. RNases are ubiquitous and general precautions should be followed in order to avoid the introduction of contaminating nucleases during the FFPE extraction process.

1.3. Always work with gloved hands and change gloves frequently.

1.4. Use RNase free, filtered pipette tips for pipetting whenever possible.

1.5. Use dedicated RNase free equipment, e.g. pipettes, pipette tips, gels boxes, etc.

DRAFT

1.6. Avoid using reagents, consumables and equipment that are in common use for other general lab processes.

1.7. When available, work in a separate room, fume hood or lab space.

1.8. Use plastic, disposable consumables that are certified RNase free.

1.9. Purchase reagents, such as commonly used buffers and water, that are certified RNase free. Prepare small individual aliquots of such buffers to avoid repeated transfer out of stock buffers. This lowers the risk of contaminating the stock solution.

1.10. Wipe down work surfaces with RNAseAway before starting.

2. Limitations

This protocol has been optimized to process up to 5 sections of 10 µm slices of formalin-fixed, paraffin-embedded tissue. Both FFPE scrolls and total nucleic acid should be stored at -80°C.

DRAFT

3. Workflow

Total Nucleic Acid Extraction from FFPE Tissue on Nimbus

Add **200 uL** of Lysis buffer to each decapped matrix tube.(*HN*)
↓
80°C Incubation for 120 min: One step deparaffinization and lysis. Peltier heater designed and made in-house. Quick spin.
↓
Decap the matrix tubes. (*CAPIT ALL*)
↓
Add **20 uL** of Proteinase K solution and mix 5X with max mixing volume. (*HN*)
↓
Recap the matrix tubes. (*CAPIT ALL*)
↓
65°C Incubation for O/N: Tissue digestion and nuclease deactivation. Peltier heater designed and made in-house. Quick spin.
↓
100°C Reverse Crosslinking for 120 minutes

→

4°C Incubation for 2 min: Snap chilling to solidify excess paraffin. Peltier heater designed and made in-house. Quick spin.
↓
Transfer 200 uL lysate to a 1.2 mL 96-Square Well, U-bottom block. (*HN*)
↓
Recap and discard the matrix tubes. (*CAPIT ALL*)
↓
Add **150 uL** of Bind buffer 1 to the 1.2mL block and mix 5X with max mixing volume (*HN*)
↓
Add **420uL** of Bind buffer 2 (20 uLs beads & 400uL IPA) to the 1.2mL block and mix 10X with max mixing volume (*HN*)
↓
65°C Incubation for 15 min without cover on Peltier heater designed and made in-house.

→

Move the 1.2 mL block plate on to SPRI Plate Ring Super Magnet Plate and clear beads for 15 min (*HN*)
↓
Slowly aspirate , 2X500 uL, and discard it. Do not disturb the beads to avoid bead carryover. (*HN*)
↓
Resuspend the beads in 300 uL wash buffer and mix 10X and incubate for 1 min. (*HN*)
↓
Transfer the resuspended beads on to the Super Mag plate and clear for 10 min. (*HN*)
↓
Discard the Supernatant without disturbing the beads. (HN)
↓
Off magnet, add **1000 uL of 85%** Ethanol and mix 5X to resuspend the beads. (*HN*)
↓
Transfer the 1.2 mL block onto the magnet plate and let clear for *5 min*. (HN)

→

Discard the supernatant, 1000 uL. (*HN*)
↓
2X Heated ,500uL, 90% IPA wash and 3 min incubation at **80°C** followed by on magnet for 5 min .
↓
Off magnet, resuspend the beads 750 uL of 85% ethanol. Mix 5X and transfer onto the magnet for 5 min (*HN*)
↓
Air dry the beads for 10 min, and resuspend in 60 uL of hot, **70°C** EB. Mix 10X and incubate for 3 min .
↓
Transfer onto the magnet for 2 min for clearing (*HN)*
↓
Transfer the superantant into a new 96 well AB1000 plate.(*HN)*

Note: Temperatures listed above are specific to the Peltier heaters designed in-house.

Non Controlled Version
**Note: Controlled Versions of this document are subjected to change without notice*

DRAFT

4. Day 1 – Lysis and Proteinase K Addition

4.1. Wipe down the workbench, small equipment, ice bucket and the Nimbus deck with RNAse Zap. Lay down new bench coat.

4.2. Take out bottle of Lysis Buffer and Wash Buffer stored at room temperature

4.3. Proteinase K (40mg/mL) Prep: Add 2.3 mL PK Buffer directly to the PK vial Write the date of assembly on the vial. Mix well by gently shaking the vial. Store the PK solution at -20°C when not in use.

4.4. Spin down the FFPE scrolls in a matrix block for full speed, **4000rmp , for 2 min**.

4.5. Use the CAPIT-ALL instrument to Decap the matrix block and refer to BSCore_WorkInst.0018. Leave the lids in the Decapper and cover the tubes with Edge tape. Return to the Nimbus and start the lysis step. If desired, for partial plates, use the 8-channel decapper and store the lids in a Storplate_96V in their corresponding wells.

4.6. Turn on a Peltier on the bench to 80°C fitted with the **matrix tube adapter**.

4.7. Log into the Nimbus laptop and start the Hamilton Run Control program. Click "File -> Open" and choose the "Production"folder. Choose "FFPE Extraction v1.0" and open "FFPE Extraction v1.0.med".

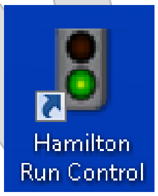

Figure 1 - Run control desktop icon

4.8. Click the "Start" button. Choose "Lysis Buffer Addition". Prepare the following reagent:

**Solution**	**Volume/well or Volume / Channel**	**Plate Type**
Lysis Buffer	5 ml/channel	Low 12 channel reservoir

DRAFT

4.9. Fill matrix plate rack with dummy tubes for less than full columns. Follow the prompts on the Nimbus to see the deck layout.

4.10. Recap Matrix tubes and vortex on the Bioshake XP at 1700 RPM for 1 minute.

4.11. Quick spin the tubes and place on the 80°C Peltier for 2 hours.

4.12. After the two hour incubation, remove the tubes from the Peltier and quick spin. Set the Peltier to 65°C fitted with the **matrix tube adapter**.

4.13. Click "File -> Open" and choose the "Production" folder. Choose "FFPE Extraction v1.0" and open "FFPE Extractionv1.0.med."

4.14. Click the "Start" button. Choose "PK Addition". Prepare the following reagent:

Solution	Volume/well or Volume / Channel	Plate Type
PK enzyme solution (if none already made up: 2.3ml PK Buffer into desiccated enzyme bottle)	25ul/well	AB1000

4.15. Decap the matrix tubes and place tape seal under all tubes. Be careful not to spill or cross contaminate the sample. A kimwipe or backside of a new tape seal can be used to push the tubes down from the top onto the bottom tape seal.

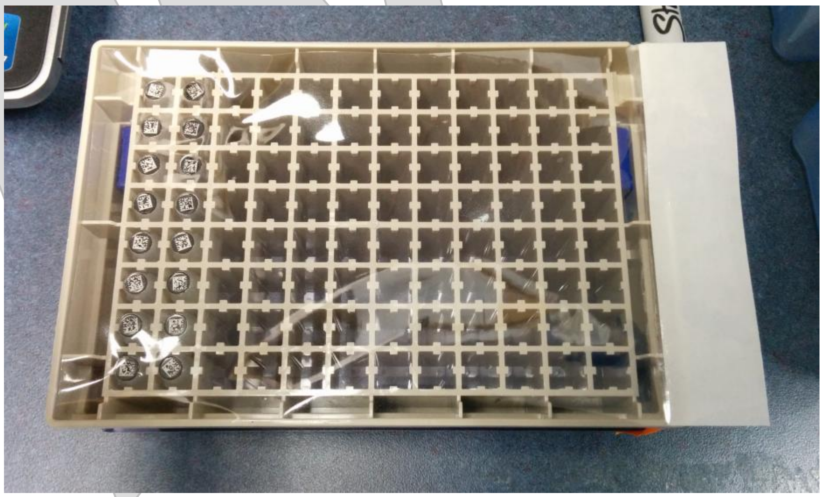

Figure 2 - Tape Seal Placed on Bottom of Matrix Tubes

4.16. After PK enzyme addition, remove tape seal from the bottom of the Matrix tubes, recap, and vortex on the Bioshake XP at 1700 RPM for 1 minute.

4.17. Quick spin the tubes and place on the 65°C Peltier overnight with the **matrix tube adapter.**

5. Day 2 – Bead Cleanup/Extraction

5.1. Wipe down the workbench, small equipment, ice bucket and the Nimbus deck with RNAse Zap. Lay down new bench coat.

5.2. An hour before you start, set a second Peltier to 100°C with the matrix adapter and reverse crosslink for two hours (note the actual temperature measured is 90°C which is intended.).

5.3. Prepare the reagents as follows:

**Solution**	**Preparation Required**	**Volume/well or Volume/channel**	**Plate Type**
85% Ethanol	For less than 2 columns: 42.5 ml of 100% Ethanol + 7.5 ml DEPC water =50ml Total<br>For up to full plate: 159.8 ml of 100% Ethanol + 28.2 ml DEPC water = 188 ml Total	Use All	Deep well reservoir
90% IPA	For less than 3 columns: 45 ml of IPA + 5ml DEPC water = 50ml Total<br>For up to full plate: 104.4 ml of IPA +11.6 ml DEPC water = 116 ml Total	Use all	Deep well reservoir

Wash Buffer (with 100% IPA added 1:1 ratio)	See Instructions on bottle, If not done so, 100% IPA should be added in 1:1 ratio	5ml/channel	Low 12 channel reservoir
Bind Buffer 1		5 ml/ channel	Low 12 channel reservoir
Qiagen Elution Buffer		100ul/well	Abgene 1000
Bind 2 Beads Solution	Make master mix for multiple samples, each reaction (with 10% for pipetting error) requires:<br>22ul Beads + 440ul 100%IPA, see Appendix Table 2	420ul/ well	AB1127

5.4. After the reverse crosslinking, perform a quick spin on the Matrix tubes

5.5. Immediately place the Matrix Tubes in ice to snap cool the tubes.

5.6. When all reagents are ready, decap the Matrix tubes and place tape seal under the tubes again.

5.7. Run the "FFPE Extraction" protocol on the Nimbus. Choose the "Bead Cleanup" step. Follow the plate layout on the Nimbus: The first picture depicts the bottom positions for layered labware , the second depicts the top positions for layered labware, and the last includes all the other positions and top positions. Be sure to use the **AB1127 peltier adapter.**

5.8. Follow the instructions given during the program.

NOTE: Do NOT put anything on top of the MultiMACS, as this can cause a crash when the Nimbus head goes to pipette into the waste plate!

5.9. Once the program is complete, label and seal the destination plate with tape seal on ice for QC or seal with foil tape for storage at --80°C .

DRAFT

5.10. Clean up the deck and discard labware. Don't forget to take the matrix tubes and holder back to the Capit-All to retrieve the lids which are still inside the instrument. Follow the instructions in the protocol to recap the tubes, then remove the holder from the instrument and shut the Capit-All down. Discard the matrix tubes.

5.11. Remove the bench coat. Wipe down the workbench, Nimbus, and MultiMacs 96 area, small equipment, and ice bucket with RNAse Zap. Place any used racks in the bucket of diluted bleach.

5.12. Return unused stock reagents back into storage at room temperature, 4ºC or -20ºC, as appropriate.

5.13. Ensure that all equipment is turned off before leaving.

6. Sample QC

6.1. FFPE total NA needs to quantified using Quant-it or Qubit assay.

6.2. If we are processing more than 12 samples, Quant-it assay is desired. Since FFPE total NA yield is quite variable, 3 dilutions should be made on the Janus and Quant-it QC following the 96-well DNA Quantification using the dsDNA Quant-iT High Sensitivity Assay Kit and VICTOR^{3}V must be run.

DRAFT

Appendix 1:

Reagent Preparation

Table 1. Day 1 and Day 2 Reagents

Reagent	Volume/Rxn (ul)	Volume/8 Rxns (uL)	Volume/96 Rxns (uL)	Comments
Lysis Buffer	200	1600	19200	Pour at least 20 mLs + desired volume. Use a pipet aid to aspirate the remainder and dispense it into a clearly labelled sterile 50 mL falcon tube and seal with parafilm.
Proteinase K	20	200	2000	DV required is 5uL/well. Dispense 25uL and quick spin. Keep on ice.
Bind Buffer 1	150	1200	14400	Minimum volume required is 3 mLs/Column, so prepare at least 3 mLs. If performing full plate prepare 36mLs. Use a Gilson P1000 to aspirate the unused Lysis buffer and pour into a sterile clearly labelled 15 mL Falcon tube for future use.
Wash Buffer (1:1 ratio formula, IPA:Wash buffer)<br><br>Follow instructions on the bottle to add appropriate amount of IPA. Option: Prepare at least 30mLs/run.	300	2400	28800	Prepare at least 30 mLs. Pour the unused back into a clearly labelled sterile 50 mL Falcon tube and seal with Parafilm. Unused wash buffer can be stored at room temperature for future use.
2 X 85% Ethanol Washes	1750	14000	168000	Deep Well Reservoir requires at least 20mLs DV. Prepare as much as you need in Falcon tubes and discard the remainder.
2 X IPA Wash (90% IPA)	1000	8000	96000	Deep Well Reservoir requires at least 20mLs DV. Prepare as much you need a falcons tubes and discard the remainder.

DRAFT

Qiagen Elution Buffer	60	480	5760	Need to aliquot at least 100uL/well for hot elution step. Use a multichannel or Gilson Repeater Pipet to dispense EB into an Abgene1000 96 well plate.

Bind 2 buffer: Bind 2 buffer is a mixture of pure beads and 100% IPA. See the table below:

Table 2. Bind 2 Prep

Bind 2 Preparation	Vol/ rxn (uL)	Vol/ 8 rxns (uL)	Vol/ 96rxns (uL)	Comments
Beads	20	176	2112	Prepare 10% Extra for pipetting inaccuracies. Aliquot 420uL of Bind 2 in as many wells as desired based on the plate layout.
100% IPA	400	3520	42240	
Bind 2: 20uL of Beads + 400 uL IPA.<br>1. **Beads**: 20uLX8+10% DV= 176uL.<br>2. **100% IPA**: 400uLX8+10% DV=3520uL.	420	3696	44352	

DRAFT

Appendix 2: LIMS

1. Automated FFPE Total NA Extraction:
    a. Discard the tissue / cell sample in LIMS.
2. DNA QC protocol
3. If constructing RNA Seq libraries, aliquot out the amount of total nucleic acid required, redefine aliquots to new library IDs
4. Create Bioanalyzer run / Caliper run: enter the dilution factor, concentration (ng/uL) and RIN / RQS. Track protocol as Agilent 2 / Caliper Total RNA QC.

DRAFT
