# Supplementary material for: Automated high throughput nucleic acid purification from formalin-fixed paraffin-embedded tissue samples for next generation sequence analysis
Source: PLoS One. 2017 Jun 1;12(6):e0178706. doi: 10.1371/journal.pone.0178706 (PMC5453589; doi:10.1371/journal.pone.0178706)
Supplement: S2 File — (PDF) [file pone.0178706.s013.pdf]

Non Controlled Version
**Note: Controlled Versions of this document are subjected to change without notice*

OFFICIAL

FFPE Genome 96-well Library Construction for Illumina Sequencing

I. Purpose

To provide specific guidelines for Pipeline Version 2, automated 96-well FFPE (formalin-fixed paraffin-embedded) DNA library construction for Illumina Paired-End Sequencing.

II. Scope

All procedures are applicable to the BCGSC Library core and Library TechD groups.

III. Policy

This procedure will be controlled under the policies of the Genome Sciences Centre, as outlined in the Genome Sciences Centre High Throughput Production Quality Manual (QM.0001). Do not copy or alter this document. To obtain a copy see a QA associate.

IV. Responsibility

It is the responsibility of all personnel performing this procedure to follow the current protocol. It is the responsibility of the Group Leader to ensure personnel are trained in all aspects of this protocol. It is the responsibility of Quality Assurance Management to audit this procedure for compliance and maintain control of this procedure.

V. References

Reference Title	Reference Number
Sample Preparation for Paired-End Sample Prep Kit from Illumina	Version 1.1 (from Prep Kit)

VI. Related Documents

Document Title	Document Number
96-well PCR-enriched Library Construction for Illumina Sequencing	LIBPR.0119
96-well DNA Quantification using the dsDNA Quant-iT high sensitivity assay kit and VICTOR^{3}V	LIBPR.0108
Operation of Covaris LE220	LIBPR.0097
Operation and Maintenance of the Agilent 2100 Bioanalyzer for DNA samples	LIBPR.0017
Operation and Maintenance of the Caliper Labchip GX for DNA	LIBPR.0051

Non Controlled Version

**Note: Controlled Versions of this document are subjected to change without notice*

Document Title	Document Number
samples using the High Sensitivity Assay	
Operation of the Invitrogen Egel iBase Power System	LIBPR_WORKINST.0012
Quantifying DNA samples using the Qubit Fluorometer	LIBPR.0030

VII. Safety

All Laboratory Safety procedures will be complied with during this procedure. The required personal protective equipment includes a laboratory coat and gloves. See the material safety data sheet (MSDS) for additional information.

VIII. Materials and Equipment

Name	Supplier	Number: #	Model or Catalogue #	
NEBNext FFPE End Repair Kit GSC	NEB	E6615B-GSC		✓
NEB Paired-End Sample Prep Premix Kit – A Tail	NEB	E6876B-GSC		✓
NEB Paired-End Sample Prep Premix Kit – Ligation	NEB	E6877B-GSC		✓
Phusion Hotstart	Fisher	F540L		✓
Fisherbrand Textured Nitrile gloves – various sizes	Fisher	270-058-53		✓
Ice bucket – Green	Fisher	11-676-36		✓
DNA *AWAY*	Molecular BioProducts	21-236-28		✓
Gilson P2 pipetman	Mandel	GF-44801	✓	
Gilson P10 pipetman	Mandel	GF-44802	✓	
Gilson P20 pipetman	Mandel	GF23600	✓	
Gilson P200 pipetman	Mandel	GF-23601	✓	
Gilson P1000 pipetman	Mandel	GF-23602	✓	
Diamond Filter tips DFL10	Mandel Scientific	GF -F171203		✓
Diamond Filter tips DF30	Mandel Scientific	GF-F171303		✓
Diamond Filter tips DF200	Mandel Scientific	GF-F171503		✓
Diamond Filter tips DF1000	Mandel Scientific	GF-F171703		✓
Galaxy mini-centrifuge	VWR	37000-700	✓	
VX-100 Vortex Mixer	Rose Scientific	S-0100	✓	
Black ink permanent marker pen	VWR	52877-310		✓
Foil Tape, 3" x 60yrds, 12rolls/case	General Fasteners	34000740		✓
Adhesive Foil for 96 well plate	VWR	60941-126		
Eppendorf BenchTop Refrigerated Centrifuge 5810R	Eppendorf	5810 R	✓	✓
Bench Coat (Bench Protection Paper)	Fisher	12-007-186		✓
Small Autoclave waste bags 10"X15"	Fisher	01-826-4		✓
IKA Works Vortexer	Agilent	MS2S9-5065-4428	✓	
22R Microfuge Centrifuge	Beckman	22R Centrifuge	✓	
Peltier Thermal Cycler	MJ Research	PTC-225	✓	
Power Supply, LVC2kW, 48VDCV	Tyco Electronics	RM200HA100	✓	
P165B Tips, sterile, 10 racks of 96/box	Ultident	24-FXF-180-LRS		✓
P50 (Universal) Tips, Presterile with Barrier, 50ul, 96/rack, 10racks/case, CS960	Beckman	CABKA21586		✓
P20B Tips, sterile, 10 racks of 96/box	Ultident	24-FXF-20-LRS		✓
Plate, 96-Well reservoirs, diamond-bottom, Low-Profile	Ultident	24-RES-SW96-LP		✓

Non Controlled Version
**Note: Controlled Versions of this document are subjected to change without notice*

Plate, 384-Well reservoirs, diamond-bottom, Low-Profile	Ultident	24-RES-SW384-LP		✓
Biomek FX Liquid Handling System	Beckman	Biomek FX	✓	
Eppendorf Benchtop Centrifuge	Eppendorf	5810 R	✓	
70% Ethanol	In house	N/A	N/A	N/A
Qiagen Buffer EB – 250ml	Qiagen	19086		✓
UltraPure Distilled Water	Invitrogen	10977-023		✓
PCR Clean-DX	Aline Biosciences	C-1003-450		✓
AB1000 96-well 200µl PCR plate	Fisher	AB1000		✓

IX. Introduction and Guidelines

1.0 General Guidelines

1.1 This protocol is designed to work with a minimum of 100ng FFPE genomic DNA or 100ng FFPE total nucleic acid in a maximum volume of 62µL. Normalize the input for a plate as much as possible.
1.2 Ensure proper personal protective equipment is used when handling sample plates, reagents and equipment. Treat everything with clean PCR techniques.
1.3 Wipe down the assigned workstation, pipetman, tip boxes and small equipment with DNA *AWAY*. Ensure you have a clean working surface before you start.
1.4 Pre-PCR and Post-PCR work should be performed on the 5th Floor and 6th floor respectively.
1.5 Acronyms: BC refers to Bead Clean.
1.6 Discuss with the APC/PC/designated trainer the results of every QC step. Report and record equipment failures and/or malfunctions and variations in reaction well volumes.

2.0 General Plate Guidelines

2.1 Use AB1000 plates for all steps.
2.2 Up to 3 plates can be processed at a time by one technologist using Biomek FX.
2.3 To avoid cross-well contamination, reaction plates should never be vortexed and plate seals should never be re-used. Use Biomek FX for mixing.
2.4 Use Qiagen tape seals for short term storage, VWR aluminum foil for Tetrad incubations and PCR, and foil tape for long term storage.
2.5 After completion of every incubation step, quick spin the plate(s) at 4ºC for 1 minute at 2000g.
2.6 Sample plates can be stored at -20ºC overnight after every step except post "A" addition. **"A" addition and adapter ligation reactions should be set up on the same day.**

3.0 Positive and Negative Controls

3.1 The positive control template to be used for this protocol is mouse FFPE DNA, extracted from tissue using Qiagen's Allprep DNA/RNA FFPE kit.

OFFICIAL

Non Controlled Version
**Note: Controlled Versions of this document are subjected to change without notice*

3.2 The negative control template to be used for this protocol is Qiagen Elution Buffer. This control will ensure the absence of background products that result from the library construction process.
3.3 The negative and positive controls will be added to each plate right before shearing according to the plate layout provided by the APC/PC/designated trainer.

4.0 General Brew Preparation Guidelines

4.1 Double check the QA release and expiry date of each reagent and enzyme.
4.2 Thaw required reagents and place them on ice. Enzymes should be left in the freezer until ready to use.
4.3 Reagents and enzymes should be well mixed, the former by pulse-vortexing and the latter by gentle flicking. After mixing, quick spin down in a mini-centrifuge.
4.4 Once prepared, all brews should be well mixed by gentle, repeated pulse-vortexing to ensure equal distribution of all components and thus uniformity of enzymatic reactions across a plate.
4.5 All reactions require the preparation of a Brew Source Plate. The Biomek FX will be used to aliquot the brew from the Brew Source Plate into the reaction plates.
4.6 All brew calculators include excess volume to account for dead volume required by the Biomek FX in the Brew Source Plate and to account for pipetting loss.

5.0 General Biomek FX Handling Guidelines

5.1 Reaction brews vary in viscosity and in order to assure accurate volume transfer it is very important to select the correct Biomek technique.
5.2 The default dead volume required by the Biomek FX in each well of a Brew Source Plate/Indexing Primer plate is 5 µL/well, regardless of the number of plates being processed (up to 3 plates).
5.3 The dead volume required by the Biomek FX in the 96-well/384-well reservoirs is 25mL.
5.4 Confirm the plate and tip box locations on the Biomek deck matches the software deck layout on the computer screen.
5.5 Ensure plate seals and tip box covers are removed before starting the Biomek program.

X. Procedure

Note: ALINE beads (PCR Clean DX) and Ampure XP beads can be used interchangeably in the magnetic bead clean up steps.

OFFICIAL

Non Controlled Version

**Note: Controlled Versions of this document are subjected to change without notice*

1. Quality and Size QC of FFPE DNA Stock

1.1 DNA in FFPE samples is prone to damage through the fixation process and prolonged storage. As a result, FFPE DNA will vary in quality and can be highly degraded. Below is a typical FFPE DNA stock E-gel profile:

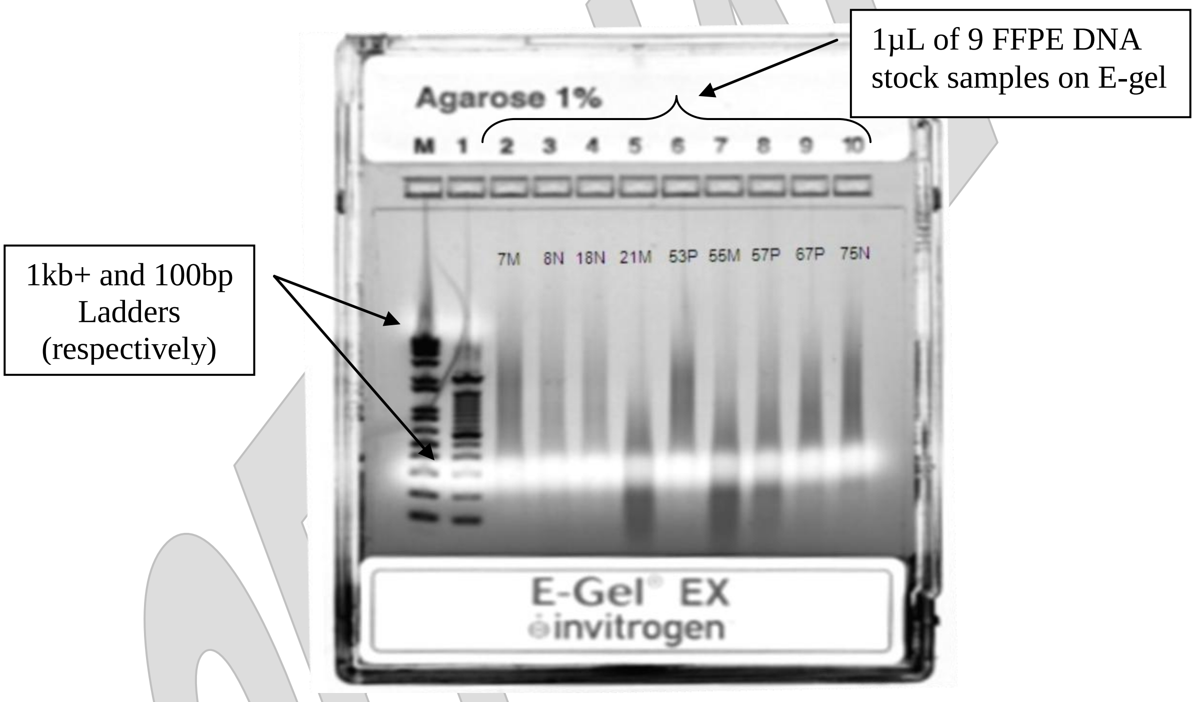

Figure 1. Stock FFPE-DNA QC on E-gel

1.2 All FFPE DNA samples will be used in library construction regardless of the profile. However, if samples have a high proportion of DNA fragments <200bp then the yields of constructed library may be much lower as the protocol is designed to remove products below 200bp.

2. Shearing

2.1. The starting material requirement for FFPE Genome library construction is 100ng FFPE DNA or 100ng FFPE total nucleic acid.

2.2. Transfer each FFPE DNA template, resuspended in 62 µL total volume of EB buffer, into a 96 microTUBE plate for shearing following the Biomek protocol:

Non Controlled Version
**Note: Controlled Versions of this document are subjected to change without notice*

Biomek: Project >LIBPR > LibraryConstruction> Run>FFPE gDNA> Shearing Setup

3. Covaris LE220 Shearing Conditions

3.1. Refer to the following instructions for shearing setup: LE220 Operation Instructions, LIBPR.0097.

3.2. LE220 Sonication Program: **Processes>LIBPR>Plate_120sec_FFPE_Modified gap**

3.3. The following shearing conditions are used for FFPE DNA:
Duty Factor - 20%
Peak Incident Power - 450W
Cycle per burst – 200
Duration – 2X 60 sec (NB: spin down plate after 60 sec. shearing and repeat)

4. Agilent HS DNA QC after shearing – Spot Check

4.1 For each 96 well plate of sheared samples, use 1µL from 11 random samples to spot check on a High Sensitivity DNA Agilent Assay according to protocol:

LIBPR.0017 Operation and Maintenance of the Agilent 2100 Bioanalyzer for DNA samples

4.2 The Agilent HS DNA assay profiles of sheared samples are dependent on the extraction method used. The profile of sheared FFPE DNA extracted by the Qiagen Allprep DNA/RNA FFPE protocol (LIBPR.0111 or LIBPR.0116) has a DNA peak height between 300 and 400bp, whereas the profile of sheared total nucleic acid products isolated using the protocol "Total Nucleic Acid Extraction from Formalin Fixed Paraffin Embedded Tissues on the NIMBUS" (TechD.0111) shows all total nucleic acid fragments, including degraded RNA fragments. In addition to the larger DNA fragments which have a peak height between 250 and 350 bp, some degraded material with a peak height of approximately 100 bp is usually observed in the Agilent profiles of total nucleic acid extracts.
Examples of each profile are shown below:

Non Controlled Version
**Note: Controlled Versions of this document are subjected to change without notice*

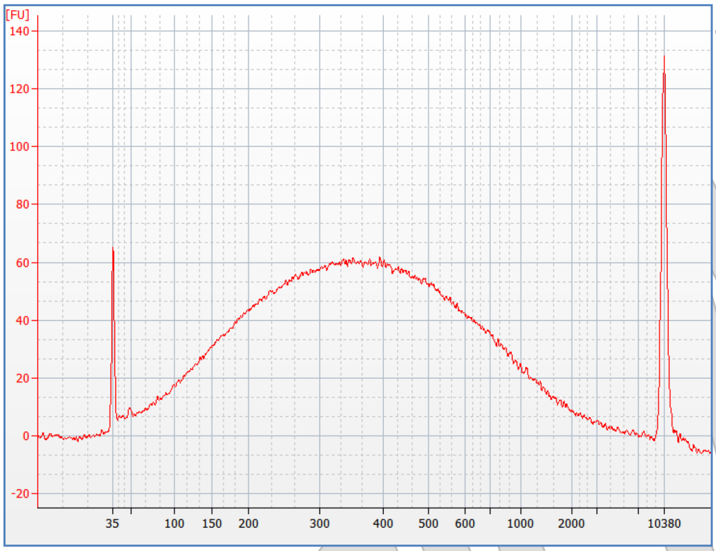

Profile of sheared Qiagen Allprep DNA extract from src91709 FFPE scrolls

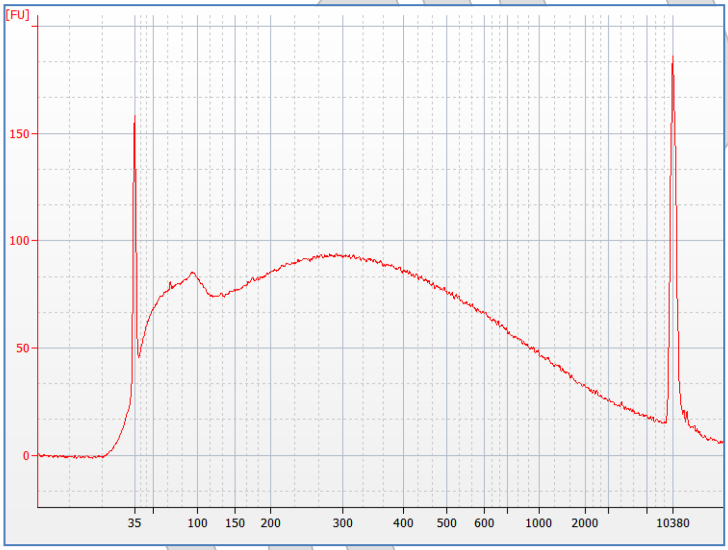

Profile of sheared FormaPure total nucleic acid extract from src91709 FFPE scrolls

4.3 Transfer sheared FFPE DNA/total nucleic acid out of the Covaris plate according to the Biomek protocol:

<u>Biomek</u>: Project >LIBPR > LibraryConstruction > Run>FFPE gDNA> **Transfer Out Of Covaris**

5. Double-Sided Beads Size Selection

5.1 To improve library quality of FFPE-derived DNA, an initial beads-based size selection step is performed after shearing and before library construction. This selects the sheared

DNA fraction approximately 200-500 base pairs in size. But more importantly, this removes smaller DNA fragments from highly degraded FFPE DNAs. If not removed in the early library construction process, these smaller fragments would otherwise cause a downward size bias in the final PCR product.

5.2 Log into Biomek FX program as follows:

> Biomek: Project >LIBPR > LibraryConstruction > Run>FFPE gDNA> **Size Select Sheared DNA**

5.3 After Biomek program completion, seal the plate and quick spin at 4ºC for 1 minute. Inspect the reaction plate for any variations in volume.

6. Combined FFPE DNA / End Repair using NEBNext FFPE End Repair

6.1. The DNA repair (FFPE damage and End repair) reactions and phosphorylation are combined in a single reaction using an enzymatic premix. The volume requirements for 1 reaction set up are as follows:

**Solution**	**1 rxn (µL)**	
Sheared, size-selected FFPE DNA	35	
Ultrapure Water	7.6	FFPE Repair Brew (15 µL)
10 x End Repair Rxn Buffer	5	
NAD+ (100x)	0.4	
PreCR/End Repair Mix (NEB)	2	
**Reaction volume**	**50**	

6.2. Generate the PreCR-Repair-Brew Mix calculator using LIMS:

> LIMS: Mix Standard Solution > **FFPE DNA Repair** > *follow the prompts* > Save Standard Solution

6.3. Obtain both the 1D large Solution/Box/Kit Label and Chemistry Label. Prepare the brew in an appropriate sized tube according to the chemistry calculator.

6.4. Using a Gilson repeat Pipetman, dispense 20 µL (includes 5 µL dead volume) of brew into each well of a Brew Source Plate. Cover with plate seal and quick spin at 4ºC for 1 minute

6.5. Log into Biomek FX Program as follows:

> Biomek: Project >LIBPR > LibraryConstruction > Run>FFPE gDNA> DNA Repair

OFFICIAL

Non Controlled Version
**Note: Controlled Versions of this document are subjected to change without notice*

OFFICIAL

6.6. The brew plate is the "Source" and the DNA plate is the "Dest." After Biomek program completion, seal the plate and quick spin at 4ºC for 1 minute. Inspect the reaction plate for any variations in volume.

6.7. Incubate FFPE DNA Repair reaction plate at 20ºC for 45 minutes.

Tetrad Program: Run > LIBCOR > PRECR/ER

7. Bead Clean Up after FFPE DNA Repair (1:1 ratio)

7.1 The 1:1 (bead:sample) ratio is essential for the removal of small FFPE fragments. The input volume for this step is 50 µL per well.

7.2 Log into Biomek FX Program as follows:

Biomek: Project > LIBPR > LibraryConstruction > Run > FFPE gDNA>Bead Clean Repaired DNA (1x)

8. Adenylation Reaction (dA Tailing)

8.1 Thaw NEB dA Tailing Reaction mix on ice, and mix by gently flicking tube.

8.2 The volume requirements for 1 reaction set up are as follows:

Solution	µL/reaction
Repaired + BC FFPE DNA	30
dA Tailing Reaction Mix (NEB)	20
**Reaction total**	**50**

8.3 Using a Gilson repeat Pipetman, dispense 25 µL of brew (contains 5 µL dead volume) into each well of a Brew Source Plate. Cover with a plate seal and quick spin at 4ºC for 1 minute.

8.4 Log into Biomek FX Program as follows:

Biomek: Project > LIBPR > LibraryConstruction > Run >FFPE gDNA> **A-Tailing**

8.5 The brew plate is the "Source" and the DNA plate(s) is the "Dest." After Biomek program completion, seal the plate and quick spin at 4ºC for 1 minute. Inspect the reaction plate for any variations in volume.

Non Controlled Version
**Note: Controlled Versions of this document are subjected to change without notice*

8.6 Incubate A-tailed reaction plate(s) at 37ºC for 30 minutes, 70ºC for 5 minutes, 4ºC for 5, hold at 4ºC

Tetrad Program: Run > LIBCOR > ATAIL

8.7 Quick spin the plate once plate is held at 4ºC and proceed immediately to ligation.

9. Adapter Ligation Reaction

9.1. Thaw the PE Adapter stock aliquot in the Tissue Culture Room laminar flowhood on the 5th floor, room 511 and immediately place on ice. Addition of PE adapter to the NEB Ligation Reaction Master Mix is made in the Blood Room laminar flowhood.

9.2. The volume requirement for 1 reaction set up is as follows:

**10pmol Ligation brew**	**µL/Reaction**
Ligation Reaction Master Mix (NEB)	21
10 µM PE Adapter	1.0
UltraPure Water	3.0
Adenylated DNA	50
**Reaction total**	**75**

10pmol Ligation Brew (25 µL)

9.3. Generate the Ligation-Brew Mix calculator using LIMS:

> LIMS: Mix Standard Solution > **Ligation_Brew_10pmol**> *follow the prompts* > Save Standard Solution

9.4. To minimize adapter-adapter ligation, work quickly on ice and proceed as follows:

9.4.1. Prepare the Ligation brew in an appropriate sized tube according to the chemistry calculator
9.4.2. Immediately after the brew is prepared, dispense 30 µL of brew (includes dead volume) into a Brew Source Plate using a Gilson repeat pipetman.
9.4.3. Cover the brew source plate with plate seal and quick spin at 4ºC for 1 minute.
9.4.4. Keep plates on ice but *proceed quickly* to the next step.

9.5. Log into Biomek FX Program as follows:

> Biomek: Project > LIBPR > LibraryConstruction > Run >FFPE gDNA> **Adapter Ligation**

Non Controlled Version

**Note: Controlled Versions of this document are subjected to change without notice*

OFFICIAL

9.6. The brew plate is the "Source" and the DNA plate(s) is the "Dest." After Biomek program completion, seal the plate and quick spin at 4ºC for 1 minute. Inspect the reaction plate for any variations in volume. Incubate Adapter Ligation reaction plate at 20ºC for 15 minutes.

Tetrad Program: RUN > LIBCOR >LIGATION

Ligation will hold at 4ºC after the 20ºC incubation. Proceed immediately to post ligation clean up once the plate is held at 4ºC.

10. Bead Clean Up after Adapter Ligation (2X, 0.9:1bead:sample)

Note: Prior to the second bead clean elution, remove the tip corresponding to the PCR brew control well from the tip box to avoid carrying over elution buffer to the PCR brew control well.

10.1. The input volume for this step is 75µL per well.

10.2. Login to Biomek FX program as follows:

Biomek: Project>LIBPR> LibraryConstruction >Run> FFPE gDNA>**Bead Clean Ligation (2x)**

11. Indexed PCR Amplification Reaction

11.1. Thaw the PE PCR primer 1.0 in the Tissue Culture Room laminar flowhood on the 5th floor, room 511 and immediately place on ice.

11.2. Thaw the Indexing Primer Plate in a working bench across from Biomek FX on the 5th floor, quick spin at 4ºC for 1 minute and immediately place on ice.

11.2.1. To keep track of freeze-thaw cycles, mark off the indexing primer plate each time the plate is <u>thawed</u> even if it is not used.
11.2.2. The maximum freeze-thaw cycles for the indexing primer plate are 5 times.
11.2.3. Ensure there is enough volume for the number of plates to be processed including the Biomek dead volume.

Non Controlled Version
**Note: Controlled Versions of this document are subjected to change without notice*

11.3. iPCR brew (minus the primers) must be made in the PCR Clean Room laminar flowhood on the 5th floor, room 510. Addition of PE PCR primer 1.0 to the brew must be made in the Tissue Culture Room laminar flowhood on the 5th floor, room 511. Addition of the Indexing Primer Plate to the reaction plate is made by the Biomek FX.

11.4. The volume requirements for 1 reaction set up, using full Ligated+BC template eluted from the previous Bead Clean Up step are as follows:

**Solution**	**1 rxn (µL)**
Adapter Ligated+BC FFPE DNA	19
5X Phusion HF Buffer	10
10mM dNTP	1
DMSO	1.5
Hot Start Phusion (2U/µL)	0.5
PE PCR primer 1.0 (25µM)	2
Ultrapure Water	12
Indexed PCR primer plate (12.5µM)	4
**Reaction volume**	**50**

PCR Brew (27µL)

11.5. Generate the PCR Brew Mix calculator using LIMS:

LIMS: Mix Standard Solution > **LibConst_IndexingPCR_Brew** > *follow the prompts* > Save Standard Solution

11.6. Obtain the 1D large Solution/Box/Kit Label and Chemistry Label. Prepare the brew in an appropriate sized tube according to the chemistry calculator. The indexing primers will be added to the Brew Source Plate using the Biomek FX.

11.7. Using a Gilson repeat Pipetman, dispense the 32 µL of brew (without indexed primers) into each well of an AB1000 Brew Source Plate. Cover with plate seal and quick spin at 4ºC for 1 minute.

11.8. Log into Biomek FX Program as follows:

Biomek: Project > LIBPR > LibraryConstruction > Run>
FFPE gDNA>**Indexed PCR**

11.9. The biomek program for iPCR setup is as follows:

11.9.1. Addition of index primers from the index primer plate to the Brew Source Plate.

Non Controlled Version
**Note: Controlled Versions of this document are subjected to change without notice*

11.9.2. Addition of the PCR Brew (containing indexed primers) from the Brew Source Plate to the reaction plate containing Ligated+BC DNA Template.

11.10. After Biomek program completion, seal the plate and quick spin at 4ºC for 1 minute. Inspect the reaction plate for any variations in volume.

11.11. Run PCR program **TSPET8** on the thermo cycler for FFPE DNA amplification. Use a rubber pad on top of the reaction plate.

TSPET8 PCR parameters:
- 98˚C 1 min
- 98˚C 15 sec
- 65˚C 30 sec
- 72˚C 30 sec
- 72˚C 5min
- 4˚C ∞

(98˚C 15 sec, 65˚C 30 sec, 72˚C 30 sec: Total of 8 Cycles PCR)

12. Bead Clean Up after PCR (2X, 0.9:1bead:sample)

12.1. The input volume for this step is 50µL per well.

12.2. Login to Biomek FX program as follows: .

Biomek: Project>LIBPR> LibraryConstruction >Run> FFPE gDNA> **Bead Clean iPCR (2x)**

13. Set-up of Fully Constructed Library QC Plate

13.1 Prepare a dilution QC plate using the following Biomek FX program:

Biomek: Project > LibraryConstruction >RUN> FFPE gDNA> **Dilute for QC**

13.2 The Biomek will generate a 1/10 dilution plate used for Caliper QC and also Quant-iT QC if libraries are to be pooled, using a solution of 0.05% Tween-20 diluted in EB.

14. Final Library HS DNA Caliper QC

14.1 For each plate, run the 10X dilution QC plate on the Caliper GX according to protocol:

Non Controlled Version
**Note: Controlled Versions of this document are subjected to change without notice*

LIBPR.0051 Operation and maintenance of the Caliper LabChip GX for DNA Samples using the High Sensitivity Assay.

14.2 Based on Caliper smear analysis for the region of DNA between 200 to 1000bp, determine and record the average base pair size and the base pair size distribution/range for each library. The profile and average gap size of individual FFPE libraries is variable, and dependent on the quality of the FFPE starting material. FFPE material containing more damage and/or smaller fragments will give rise to libraries with smaller average gap sizes. The Caliper profiles of two libraries with optimal gap sizes are shown below.

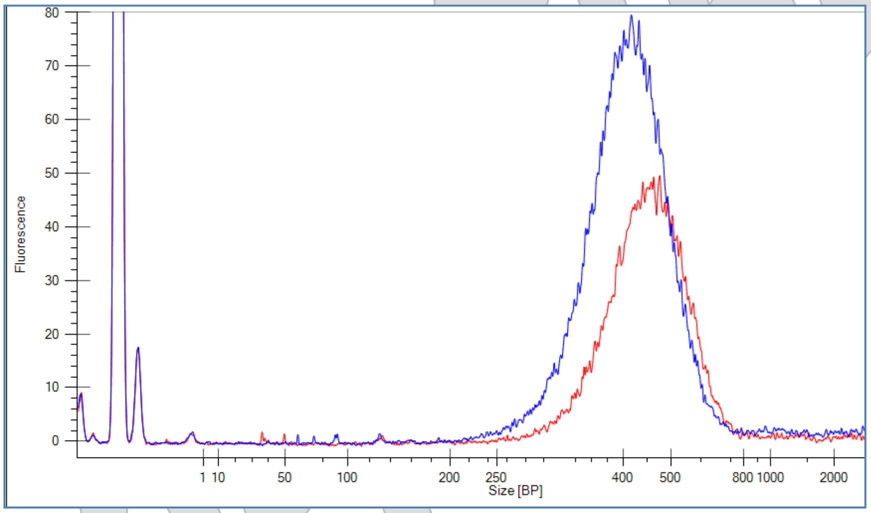

15. Quant-iT Plate-based Quantification QC for Libraries for pooling

15.1 Prior to the pooling of libraries from the 96-well plate, quantify the 10X dilution QC plate of constructed libraries using the Quant-iT assay according to the protocol:

LIBPR.0108 96-well DNA Quantification using the dsDNA Quant-iT high sensitivity assay kit and VICTOR^{3}V

16. Qubit Quantification QC for Unpooled Libraries

16.1 If libraries are to be individually sequenced, quantify the stock libraries using the Qubit assay according to the protocol:

LIBPR.0030 Quantifying DNA samples using the Qubit Fluorometer

Non Controlled Version
**Note: Controlled Versions of this document are subjected to change without notice*

17. Determination of Library Molarity

17.1 Using the average bp size results from the Caliper assay and either the Quant-iT or Qubit assay results (depending on whether libraries are to be pooled), determine the nM concentration (molarity) of each library.

18. Pooling Samples into 1.5ml Tubes on Span-8 (if needed) or Rearray Unpooled Samples into 1.5ml Tubes

18.1 Refer to the following SOP for pooling of samples:

LIBPR.0093 Span-8 Pooling of DNA Samples

19. Qubit Quantification QC for pooled libraries

19.1. Quantify pooled libraries using the Qubit assay according to the protocol:

LIBPR.0030 Quantifying DNA samples using the Qubit Fluorometer

20. Sequencing Submission

20.1. The minimum submission concentration is 15 µL of 3.0 nM library based on the Qubit quant. The maximum FFPE Genome library concentration for submission is 60 nM.

20.2. If the molarity of the library exceeds the maximum submission criteria, dilute each library to a concentration of 8nM in buffer EB supplemented with 0.1% Tween-20.

OFFICIAL

Non Controlled Version
**Note: Controlled Versions of this document are subjected to change without notice*

Appendix A: LIMS Protocol

1. Start of Plate Library Construction– IDX pipeline
2. Bioanalyzer Run-QC Category: Sonication QC
3. A-FFPE Library Construction - IDX pipeline
4. Plate_Indexed_PCR - IDX pipeline
5. Plate_PPBC_SizeSelection – IDX pipeline
6. Bioanalyzer Run/Caliper Run- QC Category: Post library construction size selection

Note: for libraries going into multiplex capture, please select "Post-PCR QC" as the QC Category instead. No need to enter attributes and please skip remaining steps.

Enter the following attributes:
   a. Library_size_distribution_bp (From Agilent or Caliper)
   b. Avg_DNA_bp_size (From Agilent or Caliper)
   c. DNA_concentration_ng_uL (From Quant-iT or Qubit)

7. If Pooling: Action: Aliquot pooling volume into a new TRA
8. If Pooling: Pooling and/or Manual Rearray into tubes – IPE pipeline
9. Final_Submission – IPE or PET pipeline

OFFICIAL

Non Controlled Version
**Note: Controlled Versions of this document are subjected to change without notice*

Appendix B: Expert SOP: 96-well FFPE Genome library construction

Step	SOP; program name	Biomek protocol: LibraryConstruction>FFPE gDNA	LIMS protocols
**Transfer DNA to Covaris plate**		> **Shearing Setup**	Start of plate library construction
**Shear DNA** (LE220)	Plate_120sec_FFPE_Modified Gap LIBPR.0097		
**QC sheared DNA:** QC 11/plate: Agilent HS DNA assay	LIBPR.0017		Bioanalyzer Run: QC type: sonication
**Transfer out of covaris plate**		> **Transfer Out Of Covaris**	
**Size Select sheared DNA**		> **Size Select Sheared DNA**	
**FFPE & End Repair**	PreCR/ER (Tetrad)	> **DNA Repair**	A-FFPE Library Construction
**Clean up End Repair (1:1)**		> **Bead Clean Repaired DNA (1X)**	
**Adenylation**	ATAIL (Tetrad)	> **A-Tailing**	
**15 minute Ligation**	LIGATION(tetrad)	> **Adapter Ligation**	
**Adapter Clean up** 2X, 0.9:1Ligation clean up		> **Bead Clean Ligation (2X)**	
**Indexing PCR**	TSPET-8 (Tetrad)	> **Index PCR**	Plate_Indexed_PCR
**Post PCR Bead Clean up** 2X, 0.9:1bead:sample clean up		> **Bead Clean iPCR (2X)**	Plate_PPBC_SizeSelection
**Dilute Libraries for QCs**		> **Dilute for QC** (20X dil.) Use for Caliper QC	
**QC Final Libraries** Agilent / Caliper HSDNA assay	LIBPR.0017 / LIBPR.0051		Bioanalyzer /Caliper Run: QC type: Post library construction size selection
**Option: Span-8 equal M pooling**	LIBPR.0093	Biomek Span-8: Project > LibCore > **Pool_Samples_from_AB1000_or_Axygen PCR 96FS_to_microfugeTubes17mm**	Action: -Aliquot to create pooling (TRA) -Rearray function to track (IX pool)
**Option: Quantify pool** Qubit HS DNA assay	LIBPR.0030		
**Submit libraries**			Final_Submission

Solutions: *FFPE DNA Repair, Ligation_Brew_40pmol; Libconst_IndexingPCR_brew*

OFFICIAL
