# Supplementary material for: Automated high throughput nucleic acid purification from formalin-fixed paraffin-embedded tissue samples for next generation sequence analysis
Source: PLoS One. 2017 Jun 1;12(6):e0178706. doi: 10.1371/journal.pone.0178706 (PMC5453589; doi:10.1371/journal.pone.0178706)
Supplement: S3 File — (PDF) [file pone.0178706.s014.pdf]

Non Controlled Version
**Note: Controlled Versions of this document are subjected to change without notice*

DRAFT

Plate-based rRNA depletion

I. Purpose

To remove cytoplasmic rRNAs (nuclear-encoded 5S, 5.8S, 18S and 28S rRNA), and mitochondrial rRNA species (12S and 16S rRNA) from Total RNA using the RNase H protocol from the New England Biolabs (NEB).

II. Scope

All procedures are applicable to the BCGSC Library Core and the Library TechD groups.

III. Policy

This procedure will be controlled under the policies of the Genome Sciences Centre, as outlined in the Genome Sciences Centre High Throughput Production Quality Manual (QM.0001). Do not copy or alter this document. To obtain a copy see a QA associate.

IV. Responsibility

It is the responsibility of all personnel performing this procedure to follow the current protocol. It is the responsibility of the Group Leader to ensure personnel are trained in all aspects of this protocol. It is the responsibility of Quality Assurance Management to audit this procedure for compliance and maintain control of this procedure.

V. References

Document Title	Document Number
NEBNext® rRNA Depletion Kit (Human/Mouse/Rat)	E6310X

VI. Related Documents

Document Title	Document Number
Operation and Maintenance of the Agilent 2100 Bioanalyzer for DNA samples	LIBPR.0017
Quantifying DNA samples using the Qubit Fluorometer	LIBPR.0030

Non Controlled Version

**Note: Controlled Versions of this document are subjected to change without notice*

Document Title	Document Number
96-well DNA Quantification using the dsDNA Quant-iT High Sensitivity Assay Kit and VICTOR[3]V	LIBPR.0108
Operation and Maintenance of the Agilent 2100 Bioanalyzer for RNA Samples	LIBPR.0018
Operation and Maintenance of the LabChipGX for RNA samples using the HT RNA Assay	LIBPR.0052
Total RNA Normalization on the Hamilton Nimbus	LIBPR.0121

VII. Safety

All Laboratory Safety procedures will be complied with during this procedure. The required personal protective equipment includes a laboratory coat and gloves. See the material safety data sheets (MSDS) for additional information.

VIII. Materials and Equipment

Name	Supplier	Number
Fisherbrand Textured Nitrile gloves - large	Fisher	270-058-53
RNAse Zap	Ambion	9780
Ice bucket – Green	Fisher	11-676-36
Wet ice	In house	N/A
RNAse free 1.5 mL eppendorf tube	Ambion	12400
Gilson P2 pipetman	Mandel	GF-44801
Gilson P10 pipetman	Mandel	GF-44802
Gilson P20 pipetman	Mandel	GF23600
Gilson P200 pipetman	Mandel	GF-23601
Gilson P1000 pipetman	Mandel	GF-23602
Mandel P200 DF200 tips	Mandel	GF-F171503
Mandel P1000 DF1000 tips	Mandel	GF-F171703
VX-100 Vortex Mixer	Rose Scientific	S-0100
200µL Rainin tips	Rainin	RT-L200F
20µL Rainin tips	Rainin	RT-L10F
200µL Pipet-Lite	Rainin	L12-200
20µL Pipet-Lite	Rainin	L12-20
1250 µL pipette tip, 96tips/rack, 480 tips/cs filter sterile	Mandel Scientific	TM-4445
Pipette-VIAFLO 8 Channel	Mandel Scientific	TM-4124
Large Kimwipes	Fisher	06-666-117

DRAFT

Non Controlled Version
**Note: Controlled Versions of this document are subjected to change without notice*

Black ink permanent marker pen	VWR	52877-310
Bench Coat (Bench Protection Paper)	Fisher	12-007-186
Small Autoclave waste bags 10"X15"	Fisher	01-826-4
DNAse1 Amplification Grade 100U	Invitrogen	18068-015
DEPC water	Ambion	9922
Mini-centrifuge	Eppendorf	5417R
Thermo Scientific 0.2mL Ultra Rigid Skirted 96-well	Thermoscientific	AB1000-150s
Deep-well, 96-well, 1.2 mL, U bottom, low pro, 50/cs	Fisher Scientific	AB1127
NEBNext® rRNA Depletion Kit (Human/Mouse/Rat)	NEB	E6310X
RNA MagClean DX	ALINE Biosciences	C-1005
Maxima H Minus First Strand cDNA Synthesis Kit	Thermo-Fisher	K165B001
Second Strand cDNA Synthesis Kit	Invitrogen	A26943
Actinomycin D 10mg/mL	In House	
Actinomycin D 5mg	MJS Biolynx	ENZGR3000005
dNTP Blend, 12.5mM with dUTP, 1mL	GeneAmp	N8080270
PCR Clean DX (ALINE Beads)	ALINE Biosciences	C-1003-450
Sterile Filtered Conductive 50**µ**L Tips in Frames	Hamilton	235979
Sterile Filtered Conductive 300**µL** Tips in Frames	Hamilton	235938
Tape Pads	Qiagen	19570
Foil Tape	VWR	60941-126
MJ Research Tetrad PTC-225 Thermal Cycler	MJ Research	8252-30-1004
G127187 DNA Engine Tetrad 2 Peltier Thermal Cycler	MJ Research	10177BD
**2100 Electrophoresis Bioanalyzer Instrument**	Agilent	G2939AA
LabChip GXII	Perkin Elmer/Caliper	124582
NIMBUS Liquid Handling Workstation	Hamilton	

IX. GENERAL GUIDELINES

1. General guidelines and input material

The following is a flow chart depicting the various steps in this protocol that will be described in detail below:

DRAFT

Non Controlled Version
*Note: Controlled Versions of this document are subjected to change without notice

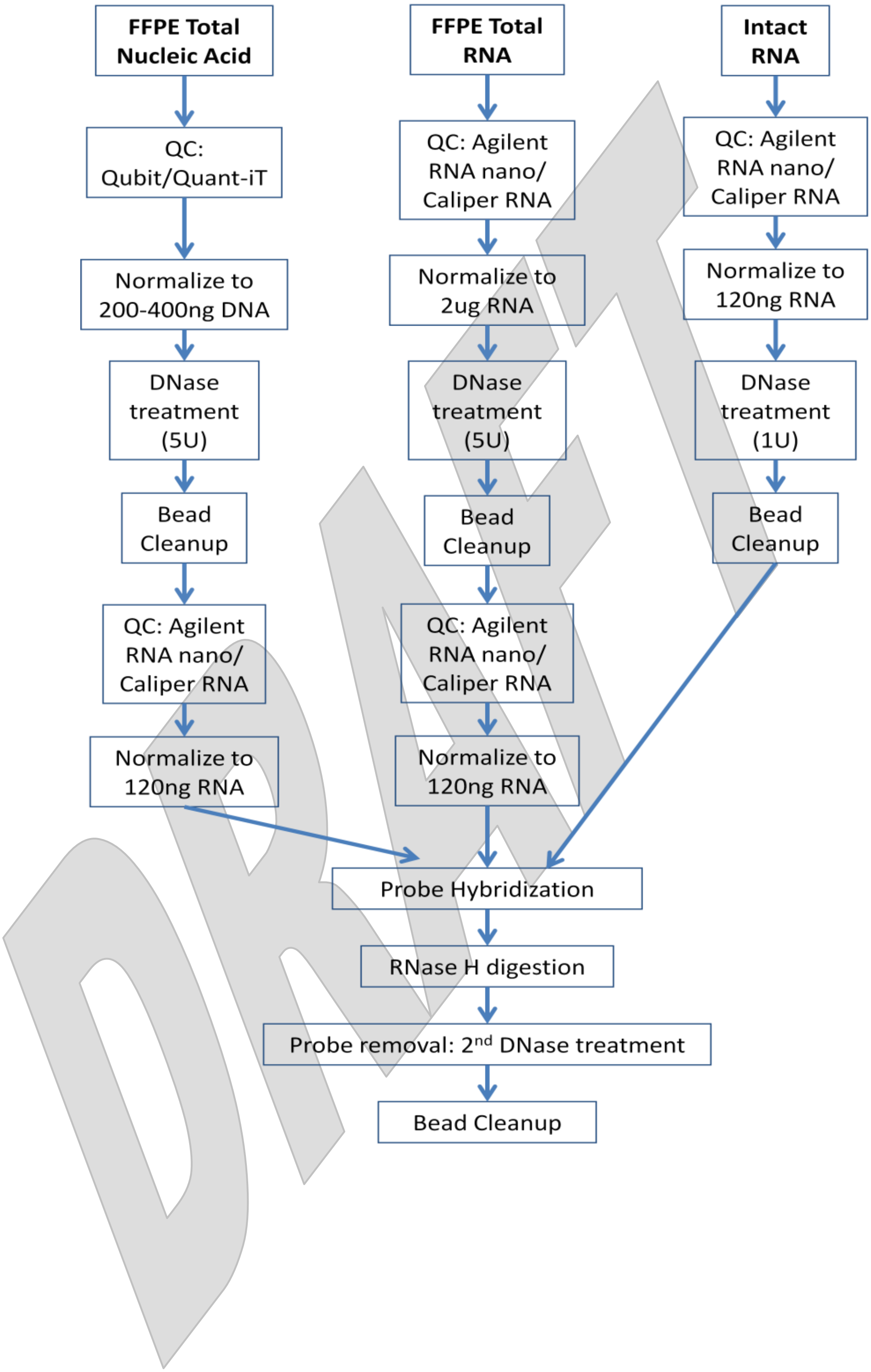

DRAFT

1.1 The recommended input material for this procedure is

Input material	Input amount
FFPE Total Nucleic Acid	200-400ng DNA
FFPE Total RNA	maximum 2ug RNA
Intact RNA	120ng RNA

For FFPE RNA, based on smear analysis on Agilent / Caliper, 100-5000 nt RNA fragments should constitute >70% of the total RNA fragments. Consult with your supervisor if the % total is <70% for this range. Input volume to be requested from collaborators is a maximum of 20µL to have suitable concentration for QC. The actual input volume for the first reaction is 35µL/well in DEPC H20 in a 96-well plate. This protocol was validated at the GSC with mouse and human RNA.

1.2 The positive control for the ribodepletion procedure is 120 ng Universal Human Reference RNA (UHR or FG031) and the negative control is DEPC H20. An additional control that should be added when cDNA synthesis is being set up is 12 ng UHR from the same immediate stock as the one that went through the ribodepletion was derived from. Make a dilution of the original stock so that you have 137 ng in 40 uL. Of this immediate stock, take 35 uL for the ribodepletion control. Take 3.5 uL for the non-ribodepleted control when you set up the cDNA reaction (to be topped up with DEPC H20). This would serve as a non-ribodepleted control. The ribodepleted UHR should give a cDNA yield of <100% of the cDNA level from non-ribodepleted UHR. Check with your supervisor if the yield is >100%. The calculations are based on agilent smear analysis (mass) within 50b-9kb range. The non-ribodepleted control will also serve as a reliable positive control for cDNA synthesis and to ensure that shearing worked later in the library construction protocol. The ribodepleted UHR control and the samples are barely detectable before iPCR.

1.3 The beads (**RNA MagClean DX**) to be used in this protocol are different from those used for other protocols as they are certified to be RNase-free.

1.4 Ensure proper personal protective equipment is used when handling sample plates, reagents and equipment. Treat everything with, and adhere to, strict RNA handling techniques.

1.5 Wipe down the assigned workstation, pipetman, tip boxes and small equipment with RNAse Zap (Ambion) followed by DEPC-treated water. Ensure you have a clean working surface before you start.

1.6 Double check the QA release and/or expiry date of each reagent and enzyme.

DRAFT

1.7 Reactions in plates should never be vortexed and plate covers are never to be re-used.

1.8 Retrieve and thaw all reagents at room temperature. Once thawed, pulse-vortex, quick spin and keep reagents on ice. Enzymes should be left in the freezer until ready to use.

1.9 Ensure the waste bag for the Nimbus is empty.

1.10 Brews are prepared and dispensed manually. Note that what is dispensed into the final brew plate is the actual volume of the specified volume for each step below without any dead volume.

1.11 Beads are manually dispensed into wells in a 1.2 ml plate (AB1127). 20 uL dead volume should be factored in, which means you need to dispense 110 uL in the final bead source plate that will be placed on the Nimbus. Ensure that you include 25 ml dead volume for 70% ethanol and DEPC H20 on top of what is required for the actual washes and elution, respectively.

1.12 The Nimbus adds sample or sample in a given reaction to the brew and bead cleanups are performed on Nimbus. Follow the prompts and lay out from the Nimbus programs to execute a particular step.

1.13 The Nimbus mixes at 80% of total volume 10 times.

1.14 Note that where it is specified that you proceed immediately to the next step, plates can be briefly placed on ice (not more than 30min) in the case of emergency. Make an active attempt to proceed as immediate as possible.

X. PROCEDURE

1. Upstream Preparation

1.1 In the case that LIMS is down, brew calculators can be located on the worksheet listed below. Enter the number of samples to be processed and print the Ribodepletion Worksheet located in:

R:\Library Core\Work Sheets and Calculators\Ribodepletion

1.2 Retrieve the plate containing Total RNA. If stored in -80°C, thaw it on ice followed by a quick spin at 4°C, 700g for 1min. Place it on ice.

DRAFT

Non Controlled Version
**Note: Controlled Versions of this document are subjected to change without notice*

2. Input QC:

2.1 For FFPE total nucleic acid, it is recommended that contaminating gDNA is first quantified using Qubit (LIBPR.0030) or Quant-IT (LIBPR.0108). RNA QC can be skipped at this stage (200-400ng gDNA should generally give more than 200ng RNA after DNase treatment). The RNA/DNA mixture entering this DNase treatment should contain <400ng gDNA. Normalization to 200-400ng gDNA should be performed using Nimbus (LIBPR.0121) or manually if deemed appropriate.

2.2 For FFPE RNA or intact RNA extracted using protocols such as Qiagen's where RNA is separated from gDNA, RNA is quantified using Agilent RNA Nano (LIBPR.0018) or Caliper RNA (LIBPR.0052) assays. Normalize intact RNA samples to 120ng and FFPE RNA to maximum 2ug using Nimbus (LIBPR.0121) or manually if deemed appropriate.

LIBPR.0121	Total RNA Normalization on the Hamilton Nimbus

3. 1st DNase I Treatment: Remove contaminating gDNA in samples

All samples should be DNase treated as follows (regardless of whether or not they were treated by collaborators). Samples should be in 35µL.

3.1 For intact RNA extracted using protocols such as Qiagen's where RNA is separated from gDNA, the reaction set up for 1 reaction is shown below. Use LIMS to generate the brew calculator for this step. If LIMS is not working, use the excel worksheet in the path above.

Solution	µL (per 1rxn)	
RNA	35	
DEPC H20	9	1st DNase Mix (15 µL)
10X DNase Buffer	5	
DNase I Enzyme	1	
**Total Reaction volume**	**50**	

For FFPE RNA or FFPE Total Nucleic acid, the reaction set up for 1 reaction is shown below:

DRAFT

Non Controlled Version
**Note: Controlled Versions of this document are subjected to change without notice*

Solution	µL (per 1rxn)
RNA	35
DEPC H20	5
10X DNase Buffer	5
DNase I Enzyme	5
**Total Reaction volume**	**50**

1st DNase Mix (15 µL) (DEPC H20, 10X DNase Buffer, DNase I Enzyme)

3.2 Make sure to mix each reagent well and quick spin before adding to the brew. Add enzyme last.

3.3 Prepare the reaction brew in a non-stick tube and check off reagents as they are added on the worksheet. Mix the brew by repeated pulse-vortexing followed by a quick spin.

LIMS Calculator (intact RNA): RBD_1st DNase_1U

LIMS Calculator (FFPE RNA or Total Nucleic acid): RBD_1st DNase_5U

3.4 Log into Nimbus Program as follows to add the RNA to 15 µL of the DNase mix:

Open file: **Production** > *toggle to workflow* > **Ribodepletion v1.2 > Ribodepletion Scheduler v1.2.wfl > Brew Additions > 1st DNase**

3.5 After Nimbus program completion, seal the plates and quick spin at 4ºC for 1 minute. Inspect the reaction plates for any variations in volume.

3.6 Incubate for 15 min at room temperature. Note: *This is NOT a safe stopping point; proceed to the next step immediately*.

3.7 Log into Nimbus Program as follows to add the RNA to 5 µL of 25mM EDTA:

Open file: **Production** > *toggle to workflow* > **Ribodepletion v1.2 > Ribodepletion Scheduler v1.2.wfl > Brew Additions > EDTA**

3.8 After Nimbus program completion, seal the plates and quick spin at 4ºC for 1 minute. Inspect the reaction plates for any variations in volume.

DRAFT

Non Controlled Version
**Note: Controlled Versions of this document are subjected to change without notice*

3.9 In the Tetrad thermocycler, incubate the plate at 65˚C for 10 min. During incubation, dispense 110 µL of Beads per well (1.2ml plate) in preparation for the subsequent cleanup.

TETRAD: RBD > RBD_65

3.10 After incubation, remove the plate and spin it at 4°C, 700g for 1min. Note: *This is NOT a safe stopping point; proceed to the next step immediately*.

4. Post–1st DNase I treatment Bead clean up (use RNA MagClean DX beads)

4.1 The input volume for this step is 50 µL per well. Note that even though the previous reaction is in 55µL, you will be prompted to transfer 50µL. The supernatant after 1st clearing from the bead cleanup may or may not be used as miRNA fraction. The reason for transferring 50µL is to ensure consistent size selection in the case that miRNA fraction is required. Check with your supervisor if the miRNA fraction needs to be processed.

4.2 The Nimbus will perform the cleanup of the 1st DNase reaction using beads as follows:

Reaction	*Bead Vol manually dispensed (µL)	Bead Vol added by Nimbus (µL)	Bead Binding Time (mins)	Magnet Clearing Time (mins)	2X 70% EtOH* Wash Vol (µL)	Magnet Airdry Time (mins)	DEPC Elution Vol (µL)	Elution time (mins)	Magnet Elution time (mins)	Transfer Vol (µL)
1st DNase Reaction	110	90	15	7	180	5	15	3	2	15

*Must be at Room Temp for a minimum of 30mins before usage; failure to do so would result in a decrease in yield

4.3 Log into Nimbus Program as follows:

Open file: **Production** > *toggle to workflow* > **Ribodepletion v1.2 > Ribodepletion Scheduler v1.2.wfl > Bead Clean > RNA Bead Cleanup v1.1 > RBD Post 1st DNase**

If the miRNA fraction is to be kept, click on the box when asked if you would like to keep the supernatant. Follow the prompts to place a 1.2mL Deep-well plate in the destination position. Once supernatant has been transferred, remove plate, seal with foil tape, label as 'supernatent', and store at -80°C. Continue with bead clean as instructed by the Nimbus.

Non Controlled Version
**Note: Controlled Versions of this document are subjected to change without notice*

This is a safe stopping point. If needed, the plate containing the bead cleaned RNA can be stored at ***-80°.***

4.4 For FFPE Total Nucleic Acid and FFPE RNA, DNase-treated RNA should be quantified using Agilent RNA Nano (LIBPR.0018) or Caliper RNA (LIBPR.0052). Skip this QC for intact RNA. Based on smear analysis, 100-5000 nt RNA fragments should constitute >70% of the total RNA fragments. Consult with your supervisor if the % total is <70% for this range. Confirm with your supervisor on which assay to use.

4.4.1 For Caliper QC on Standard Assay, use 2 µL of sample and 46 µL of made up HT RNA Caliper buffer.

4.4.2 For Caliper QC on HiSens Assay, use 2 µL of sample diluted with 4 µL DEPC water (6 µL total) and 19 µL of made up HT RNA Caliper buffer. Also, select "AB1000_2.5mm" as the total volume is lower for the Caliper plate. For Caliper QC:

LIBPR.0052 Operation and Maintenance of the LabChipGX for RNA samples using the HT RNA Assay

For Caliper plate setup on the Nimbus:

Open file: **Production** > *toggle to workflow* > **Ribodepletion v1.2 > Ribodepletion Scheduler v1.2.wfl > Caliper Transfer** > **Caliper Transfer v1.0** > **Standard** *or* **High Sensitivity**

4.5 DNase-treated RNA from FFPE Total nucleic Acid and FFPE RNA should be normalized to 120ng at this stage using Nimbus according to LIBPR.0121.

5. Hybridization: Annealing of rRNA probes to target rRNAs

Note: all reagents for steps 5, 6, and 7 including the DNAse I in Step 7 are from the NEBNext rRNA depletion kit.

DRAFT

Non Controlled Version
**Note: Controlled Versions of this document are subjected to change without notice*

5.1 The reaction set up for 1 reaction is as follows:

**Solution**	**1 rxn (µL)**	
DNase-treated RNA	12	
Depletion solution (probes)	1	Hyb Mix (3 µL)
Hybridization Buffer	2	
**Total volume**	**15**	

5.2 Make sure to mix each reagent well and quick spin before adding to the brew.

5.3 Prepare the brew and check off reagents as they are added. Mix the brew by repeated pulse-vortexing followed by a quick spin.

LIMS Calculator: RBD_Hybridization

5.4 Log into Nimbus Program as follows to add the RNA to 3 µL of the Hyb mix:

Open file: **Production** > *toggle to workflow* > **Ribodepletion v1.2 > Ribodepletion Scheduler v1.2.wfl > Brew Additions > Hybridization**

5.5 After Nimbus program completion, seal the plates and quick spin at 4ºC for 1 minute. Inspect the reaction plates for any variations in volume.

5.6 In the MJ Research Gradient Tetrad thermocycler, incubate the plate as follows:

TETRAD: RNAHYB

- 95˚C 2 min
- 95˚C , -0.1˚C at 1 sec down to 22˚C (730 cycles)
- 22˚C 5min

Attend this immediately after the incubation is done (within 30min).

5.7 After thermo-cycler program is finished, remove the plate and spin it at 4°C, 700g for 1min. Note: *This is NOT a safe stopping point; proceed to the next step immediately*.

DRAFT

Non Controlled Version
**Note: Controlled Versions of this document are subjected to change without notice*

6. RNase H digestion: Degradation of RNA of in rRNA/ DNA probe hybrid

6.1 The reaction set up for 1 reaction is as follows:

Solution	1 rxn (µL)
RNA in hyb reaction	15
Nuclease free water	1
RNase H Reaction Buffer	2
RNase H	2
**Total volume**	**20**

RNase H mix (5 µL) (Nuclease free water, RNase H Reaction Buffer, RNase H)

6.2 Make sure to mix each reagent well and quick spin before adding to the brew.

6.3 Prepare the brew and check off reagents as they are added. Mix the brew by repeated pulse-vortexing followed by a quick spin.

LIMS Calculator: RBD_ RNaseH

6.4 Log into Nimbus Program as follows to add the RNA to 5 µL of the RNase H mix:

Open file: **Production** > *toggle to workflow* > **Ribodepletion v1.2 > Ribodepletion Scheduler v1.2.wfl > Brew Additions > RNaseH**

6.5 After Nimbus program completion, seal the plates and quick spin at 4ºC for 1 minute. Inspect the reaction plates for any variations in volume.

6.6 In the Tetrad thermocycler, incubate the plate at 37˚C for 30 min (Heated Lid 47˚C).

TETRAD: RBD>RBD_37

6.7 After incubation, remove the plate and spin it at 4°C, 700g for 1min. Note: *This is NOT a safe stopping point; proceed to the next step immediately*.

DRAFT

Non Controlled Version
**Note: Controlled Versions of this document are subjected to change without notice*

7. 2nd DNase I treatment: Degradation of rRNA DNA probes

7.1 The reaction set up for 1 reaction is as follows (all reagents for this reaction should be from the Ribodepletion kit E6310X).

Solution	1 rxn (µL)	
RNA in RNase H reaction	20	
Nuclease free water	22.5	DNase mix (30 µL)
DNase Buffer (from NEBNext Kit)	5	
DNase I (from NEBNext Kit)	2.5	
**Total volume**	**50**	

7.2 Make sure to mix each reagent well and quick spin before adding to the brew.

7.3 Prepare the brew and check off reagents as they are added. Mix the brew by repeated pulse-vortexing followed by a quick spin.

LIMS Calculator: RBD_ 2nd DNase

7.4 Log into Nimbus Program as follows to add the RNA to 30 µL of the DNase mix:

Open file: **Production** > *toggle to workflow* > **Ribodepletion v1.2 > Ribodepletion Scheduler v1.2.wfl > Brew Additions > 2nd DNase**

7.5 After Nimbus program completion, seal the plates and quick spin at 4ºC for 1 minute. Inspect the reaction plates for any variations in volume.

7.6 In the Tetrad thermocycler, incubate the plate at 37˚C for 30 min (Heated Lid 47˚C). During incubation dispense 110 µL of Beads per well (1.2ml plate) in preparation for the subsequent cleanup.

TETRAD: RBD>RBD_37

7.7 After incubation, remove the plate and spin it at 4°C, 700g for 1min. Note: *This is NOT a safe stopping point; proceed to the next step immediately.*

DRAFT

Non Controlled Version
**Note: Controlled Versions of this document are subjected to change without notice*

8. Post–2nd DNase treatment Bead clean up (use RNA MagClean DX beads)

8.1 The input volume for this step is 50µL per well.

8.2 The Nimbus will perform the cleanup of the 2nd DNase reaction using beads as follows:

Reaction	*Bead Vol manually dispensed (µL)	Bead Vol added by Nimbus (µL)	Bead Binding Time (mins)	Magnet Clearing Time (mins)	2X 70% EtOH* Wash Vol (µL)	Magnet Airdry Time (mins)	DEPC Elution Vol (µL)	Elution time (mins)	Magnet Elution time (mins)	Transfer Vol (µL)
2nd DNase Reaction	110	90	15	7	180	5	37	3	2	37

*Must be at Room Temp for a minimum of 30mins before usage; failure to do so would result in a decrease in yield

8.3 Log into Nimbus Program as follows:

Open file: **Production** > *toggle to workflow* > **Ribodepletion v1.2** > **Ribodepletion Scheduler v1.2.wfl > Bead Cleans > RNA Bead Cleanup v1.1 > RBD Post 2nd DNase**

This is a safe stopping point. If needed, the plate containing the bead cleaned RNA can be stored at -80°.Otherwise, proceed to cDNA synthesis as described below. Note that brew additions and bead clean ups during cDNA synthesis are performed on Nimbus. The bead to reaction ration is 1.8 to 1 (as opposed to 2:1 in SSTRA RNA Seq pipeline) to be consistent with upstream cleanups in this SOP. Remember to include the 12ng UHR RNA for non-ribodepleted control while setting up the 1st strand cDNA synthesis reaction.

9. 1st strand cDNA Synthesis

9.1 The reaction set up for one reaction is as follows:

DRAFT

Non Controlled Version
**Note: Controlled Versions of this document are subjected to change without notice*

Solution	1 rxn (µL)
Heat Denatured ribodepleted RNA	34.3
5X First Strand Buffer	10.0
10mM dNTP mix	2.5
Random Hexamers (200ng/uL)	2.0
ActinomycinD (10ug/uL)	0.2
Maxima H Minus Enzyme Mix	1.0
**Total volume**	**50**

1st Strand Mix (15.7 µL)

9.2 Make sure to mix each reagent well and quick spin before adding to the brew.

LIMS Calculator: SS_cDNA_1st_strand_Maxima_H_Minus

9.3 Prepare the brew and check off reagents as they are added. Mix the brew by repeated pulse-vortexing followed by a quick spin.

9.4 Add 12 ng of non-ribodepleted UHR RNA to the plate. Cover plate with a foil seal.

9.5 Heat denature the plate for 5 minutes at 70C. Immediately chill on ice for 1 minute and then quick spin plate prior to brew addition.

9.6 Log into Nimbus Program as follows to add 1st strand brew to heat denatured ribo-depleted RNA.

Open file: **Production** > *toggle to workflow* > **cDNA v1.1 > cDNA Scheduler v1.1.wfl > cDNA Brew Additions > 1st strand cDNA**

9.7 After Nimbus program completion, seal the plates and quick spin at 4ºC for 1 minute. Inspect the reaction plates for any variations in volume.

9.8 In the Tetrad thermocycler, incubate the plate at 25°C for 10 minutes and then 50°C for 1 hour.

TETRAD: CDNA1MAX

9.9 After completion, quick spin plate and proceed immediately to bead clean 1st strand synthesis.

Non Controlled Version
**Note: Controlled Versions of this document are subjected to change without notice*

10. Post–1st strand synthesis Bead clean up (Use PCR Clean DX Beads)

10.1 The input volume for this step is 50µL per well.

10.2 The Nimbus will perform the cleanup of the 1st strand synthesis reaction using beads as follows:

Reaction	Bead Volume to dispense (µL)	Bead* Vol by Nimbus (µL)	Bead Binding Time (mins)	Magnet Clearing Time (mins)	2X 70% EtOH* Wash Vol (µL)	Magnet Airdry Time (mins)	EB Elution Vol (µL)	Elution time (mins)	Magnet Elution time (mins)	Transfer Vol (µL)
1st strand ss-cDNA	110	90	15	7	150	5	36	3	2	35**

*must be

*Must be at Room Temp for a minimum of 30mins before usage; failure to do so would result in a decrease in yield
** The elution and transfer is set for 36µL to ensure that we'll have sufficient and consistent resulting volume of at least 35µL in the second strand cDNA synthesis reaction.

10.3 Log into Nimbus Program as follows:

Open file: **Production** > *toggle to workflow* > **cDNA v1.1 > cDNA Scheduler v1.1.wfl > Bead Clean > RNA Bead Cleanup v1.1 > 1st strand cDNA**

10.4 After Nimbus completions, remove the plate, cover and then spin it at 4°C, 700g for 1min. Note: *This is a safe stopping point. Store 1st strand cDNA at -80C overnight or proceed immediately to second strand synthesis.*

11. 2st strand cDNA Synthesis

11.1 The reaction set up for one reaction is as follows:

DRAFT

Non Controlled Version
**Note: Controlled Versions of this document are subjected to change without notice*

Solution	1 rxn (μL)
1st strand cDNA	35
5X Second Strand Buffer	10.0
Gene Amp mix with dUTP (12.5 mM)	1.5
E. coli DNA Ligase (10U/uL)	0.5
E. coli DNA Polymerase (10U/uL)	1.5
E. coli RNase H (2U/uL)	0.5
DEPC water	1.0
**Total volume**	**50**

2nd Strand Mix (15 μL)

11.2 Make sure to mix each reagent well and quick spin before adding to the brew.

LIMS Calculator: SS_cDNA_2nd_Strand

11.3 Prepare the brew and check off reagents as they are added. Mix the brew by repeated pulse-vortexing followed by a quick spin.

11.4 Log into Nimbus Program as follows to add second strand brew to 1st strand cDNA template.

Open file: **Production** > *toggle to workflow* > **cDNA v1.1 > cDNA Scheduler v1.1.wfl > cDNA Brew Additions > 2nd strand cDNA**

11.5 After Nimbus program completion, seal the plates and quick spin at 4ºC for 1 minute. Inspect the reaction plates for any variations in volume.

11.6 In the Tetrad thermocycler, incubate the plate at 16°C for 2 hours and 15 minutes.

TETRAD: CDNA_2

11.7 After thermo-cycler program is finished, remove the plate and spin it at 4°C, 2000g for 1min.
Note: ***This is a safe stopping point. If needed, the plate can be stored at -20°C, or on the tetrad overnight if there is insufficient time to take the plate out for cold storage*.**

DRAFT

Non Controlled Version

**Note: Controlled Versions of this document are subjected to change without notice*

12. Post–2nd strand synthesis Bead clean up (Use PCR Clean DX Beads)

12.1 The input volume for this step is 50µL per well.

12.2 The Nimbus will perform the cleanup of the 2nd strand synthesis reaction using beads as follows:

Reaction	Bead Volume to dispense (µL)	Bead* Vol (µL) by Nimbus	Bead Binding Time (mins)	Magnet Clearing Time (mins)	2X 70% EtOH* Wash Vol (µL)	Magnet Airdry Time (mins)	EB Elution Vol (µL)	Elution time (mins)	Magnet Elution time (mins)	Transfer Vol (µL)
2nd strand ss-cDNA	110	90	15	7	150	5	42	3	2	41 (1µL for QC)

*Must be at Room Temp for a minimum of 15mins before usage; failure to do so would result in decreased yield.

12.3 Log into Nimbus Program as follows:

Open file: **Production** > *toggle to workflow* > **cDNA v1.1 > cDNA Scheduler v1.1.wfl > Bead Clean > RNA Bead Cleanup v1.1 > 2nd strand cDNA**

12.4 After Nimbus program completion, seal the plates and quick spin at 4ºC for 1 minute. Inspect the reaction plates for any variations in volume.

13. HS Agilent QC of double stranded ss-cDNA

13.1 For quality and quantity control check of the cDNA, use 1µL of each sample or representation of samples including the UHR controls and the negative control for High Sensitivity DNA Agilent assay according to protocol: LIBPR.0017. The non-ribodepleted UHR control may be the only sample that has visible cDNA yields. All other samples should have equal or lower yields of cDNA. The ribodepleted UHR should give a cDNA yield of <100% of the cDNA level from non-ribodepleted UHR. Check with your supervisor if the yield is >100%. The calculations are based on agilent smear analysis (mass) within 50b-9kb range. The non-ribodepleted control will also serve as a reliable positive control for cDNA synthesis and to ensure that shearing worked later in the library construction protocol. The ribodepleted UHR control and the samples are barely detectable before iPCR.

DRAFT

Non Controlled Version
**Note: Controlled Versions of this document are subjected to change without notice*

Appendix A: LIMS SOP

1. Start of Plate Library Construction

2. Bioanalyzer Run / Caliper Run – if working with FFPE RNA or FFPE total nucleic acid, QC samples on Agilent after 1st DNase treatment. Create Bioanalyzer Run – QC Category: Total RNA QC

3. Ribodepletion-1st DNAse Treatment: pass barcode to supervisor

4. Ribodepletion: Use the barcode your supervisor passes to you.

5. Pass barcode to supervisor to add control for cDNA synthesis.

6. A-Strand Specific cDNA Synthesis: RNA_strategy is "strand specific" and the pipeline is RBD_1.0: Ribodepletion 1.0

7. Bioanalyzer Run – QC Category: cDNA QC

8. Pass barcode to supervisor to add controls for library construction.

DRAFT
