# Supplementary material for: Automated high throughput nucleic acid purification from formalin-fixed paraffin-embedded tissue samples for next generation sequence analysis
Source: PLoS One. 2017 Jun 1;12(6):e0178706. doi: 10.1371/journal.pone.0178706 (PMC5453589; doi:10.1371/journal.pone.0178706)
Supplement: S4 File — (PDF) [file pone.0178706.s015.pdf]

MicroRNA Library Construction using randomized adapters for Illumina Sequencing.

I. Purpose

To prepare total RNA samples for miRNA sequencing in a 96-well well plate format. Randomized adapters are expected to reduce the biased selection of miRNA species.

II. Scope

All procedures are applicable to the BCGSC Library TechD and Production Groups.

III. Policy

This procedure will be controlled under the policies of the Genome Sciences Centre, as outlined in the Genome Sciences Centre High Throughput Production Quality Manual (QM.0001). Do not copy or alter this document. To obtain a copy see a QA associate.

IV. Responsibility

It is the responsibility of all personnel performing this procedure to follow the current protocol. It is the responsibility of the Library Core Group Leader to ensure personnel are trained in all aspects of this protocol. It is the responsibility of Quality Systems Team to audit this procedure for compliance and maintain control of this procedure.

V. References

Document Title	Document Number
Preparing samples for Small RNA sequencing using the alternative v 1.5 protocol	Illumina © 2009

VI. Related Documents

Document Title	Document Number
Operation and Maintenance of the Agilent 2100 Bioanalyzer for DNA samples	LIBPR.0017
Quantifying DNA Samples using the Qubit Fluorometer	LIBPR.0030

VII. Safety

All Laboratory Safety procedures will be complied with during this procedure. The required personal protective equipment includes a laboratory coat and gloves. See the material safety data sheet (MSDS) for additional information.

VIII. Materials and Equipment

Name	Supplier	Number	Model or Catalogue #	
5’ miRNA Adapter_6N, 1uM	IDT	NA		
3’ adenylated adapter_6N, 2.5uM	IDT	NA		
RT primer, 20uM	IDT	NA		
PE 1.0 primer, 25uM	IDT	NA		
Indexed primers, 25uM	IDT	NA		
T4 RNA ligase 2, truncated (200U/uL)	NEB	M0242L		✓
50% PEG 8000	NEB	M0242L		
10X T4 RNA Ligase buffer	NEB	M0242L		
T4 RNA Ligase (5U/uL)	Ambion	AM2141		
10 mM ATP, molecular grade	NEB	9804		
RNase Out	Invitrogen	10777-109		
DEPC-treated water	Ambion	9922		
Maxima H Minus, 200U/uL	ThermoFisher	EP0753		
5X RT Buffer	ThermoFisher	EP0753		
dNTPs Mix, 10uM	Invitrogen	46-0519		
DMSO (Dimethyl sulfoxide)	Fisher Scientific	BP231-100		
5X Phusion buffer	Finnizymes	F-518		
Phusion HS high fidelity DNA pol	NEB	F-540L		
RNase Zap	Ambion	9780		
DNA*AWAY*	MBS	7010		✓
Gilson P2 pipetman	Mandel	GF-44801		✓
Gilson P10 pipetman	Mandel	GF-44802		✓
Gilson P20 pipetman	Mandel	GF23600		✓
Gilson P200 pipetman	Mandel	GF-23601		✓
Gilson P1000 pipetman	Mandel	GF-23602		✓
Neptune barrier tips 10 µl	CLP	Bt10XL		✓
Neptune barrier tips 20 µl	CLP	Bt20		✓
Neptune barrier tips 200 µl	CLP	Bt200		✓
Neptune barrier tips 1000 µl	CLP	Bt1000		✓
Galaxy mini-centrifuge	VWR	37000-700		✓
Bench Coat (Bench Protection Paper)	Fisher	12-007-186		✓
Small Autoclave waste bags 10”X15”	Fisher	01-826-4		✓
Rainin RNase free tips (1-20uL)	Rainin	RT-L10F		✓
Rainin RNase free tips (20-200uL)	Rainin	RT-L200F		✓
10x BPB/XC loading buffer	In house	N/A	N/A	N/A

100bp ladder	Invitrogen	**Cat. No. 15628-19**		✓
SybrGreen	CAMBEX	50513		✓
Dark Reader (Transilluminator)	InterSicence	DR-190M	✓	
Gel Elution buffer	In house	N/A	N/A	N/A
Spin-X Filter Columns	Costar	8160	✓	
2mL RNase/DNase free tubes				
Abgene 96-well plate		AB1000		
P450 96-well plate				
Weight boats (Ethanol, IPA dispense)				

Oligo Sequences

miRNA 3’ Adapter , 2.5µM working stock
5’ NNNNNNTGGAATTCTCGGGTGCCAAGTCG 3’, N= randomized sequence

miRNA 5’ Adapter, 1µM working stock
5’ UUCCCUACACGACGCUCUUCCGAUCUNNNNNN 3’, N= randomize sequence

RT primer for First Strand Synthesis, 20µM working stock
5’ CGACTTGGCACCCGAGAATTCCA 3’

PE 1.0 primer, 25µM working stock
5’AATGATACGGCGACCACCGAGATCTACACTCTTTCCCTACACGACGCTCTTCCGAT
CT 3’

miRNA indexed primers, 25µM working stock
CAAGCAGAAGACGGCATACGAGATCNNNNNNATCGTACGTCGACTTGGCACCCGAG
AATTCCA, underlined index sequence is unique for up to 96 samples.

IX. Procedure

1. Initial Guidelines and Input QC

1.1. All of the steps in this protocol up to and including first strand synthesis are to be carried out in the designated RNA working spaces. First strand purification and PCR set up are performed in the designated Pre-PCR spaces. Post PCR steps are performed in the designated post-PCR designated spaces.

1.2. Prepare brew reactions in the PCR clean room on the 5th floor in the designated laminar flow hood or from one-use aliquots at a designated RNA area work station.

1.3. The starting material for this procedure is total RNA or alternatively flow-through RNA collected after mRNA isolation using the MultiMACs isolation system.

1.4. Evaluate total RNA or flow through fraction on the Caliper following LIBPR.0052 or alternatively using Agilent RNA total eukaryotic RNA assay following LIBPR.0018.

1.5. This pipeline has been validated to use a minimum of 250 ng in 8 µL (37.25ng/µL) and a maximum of 1000 ng in 8 µL (125 ng/µL). For FFPE, 1000 ng total RNA amount is recommended.

1.6. Do not treat total RNA sample with DNase I prior to starting the procedure.

2. Retrieval of Reagents and Equipment Preparation

2.1. Put on clean gloves and a new disposable lab coat.

2.2. Wipe down the workbench, small equipment and ice bucket with RNase Zap. Lay down new bench coat.

2.3. Turn on a plate-based centrifuge and pre-chill to 4C. Set one tetrad to hold at 70C for denaturation steps.

2.4. Retrieve metal blocks from 4C and wipe them down with RNase Zap and place on ice.

3. Template Transfer

3.1. Retrieve the plate or tubes containing the total RNA or flow through fraction from -80C and thaw on ice. Do not remove the cover. Once thawed, quick spin the plate and then return the plate to ice.

3.2. Aliquot the starting amount to a new 96-well plate and top up any wells to 8uL with DEPC-treated water. Ensure that the plate is clearly labeled. Seal source and destination plates, the former with foil tape, the later with temporary tape seal. Return the stock plate to -80C and place the working plate on ice.

3.3. Add 1ug of FG032A Placental total RNA (positive control) and 8 uL of DEPC-treated water (negative controls) to two empty wells.

4. 3' Adapter Ligation

4.1. Retrieve an aliquot of 2.5 µM 3' adapter from -20°C storage. Allow the DNA adapter to thaw at room temperature and then return the adapter tube to ice. Aliquots are intended for one time use, do not freeze-thaw DNA adapters. Throw out any remaining adapter.

4.2. Retrieve reagents required for 3' adapter ligation. Allow PEG 8000 to reach room temperature before use. Allow buffers to reach room temperature and then return to ice. Enzymes should be stored at -20°C until required to prepare brew.

4.3. Gently vortex adapter and then quick spin. Aliquot adapter into one column of a 96-well plate and quick spin and then return to ice. Carefully dispense 2 µL of adapter to wells of an empty AB1000 plate using a multichannel pipette. Quick spin the plate and then store it on ice.

4.4. Set a tetrad to hold at 70°C and place a label on the tetrad to show that it is in use.

4.5. Gently vortex adapter and then quick spin. Dispense 2uL of adapter to empty wells of an AB1000 plate and then cover and quick spin the plate. Double check that volumes are consistent across the wells. Transfer the template to adapter and mix 10X. Quick spin plate and then return to ice.

4.6. Set a timer for two minutes and then incubate the RNA template and 3' adapter at 70°C for 2 minutes in a tetrad. Snap chill on ice for 1 minute and then quick spin plate and return to ice.

4.7. Prepare 3' ligation brew. The volume requirement for 1 reaction set up is as follows:

Solution	1 rxn (µL)
Denatured RNA+3' Adapter	10
10X T4 RNA ligase buffer	2
DEPC-treated water	1
50% PEG 8000	5
Rnase Out	0.5
T4 RNA ligase 2 (trunc)	1.5
**Reaction volume**	**20**

miRNA_3'LigBrew
(10µL)

4.8. Generate the 3'miRNALig Brew Mix calculator using LIMS:

4.9. Obtain the 1D large Solution/Box/Kit Label and Chemistry Label. Prepare the brew in an appropriate sized tube according to the chemistry calculator.

LIMS: Mix Standard Solutions > **miRNA_3'LigBrew**

4.10. Prepare brew according to the LIMS calculator. Prepare enough reactions to provide dead volume for brew preparation and dispense.

4.11. While on ice, add 10 uL of 3' ligation brew to empty wells using a single channel pipette, cover and then quick spin the plate. Use a multichannel pipette to transfer all of the template to the wells containing brew and mix 10X, ensure that no liquid is retained in the tips. Cover plate with VWR foil and then quick spin plate. Double check that volumes are even prior to starting the incubation.

4.12. Incubate plate for one hour at 22°C using the following tetrad protocol.

MIRNA>3LIG

4.13. Pre-warm PCR DX ALINE beads and 70% Ethanol during the 3' ligation protocol.

4.14. Once the protocol has completed, remove the plate from the tetrad and quick spin the plate. Double check that volumes are normal across the plate.

4.15. Proceed to 2X bead purification. This is not a safe stopping point.

5. 2X Bead Clean

5.1. This step is essential to remove excess 3' adapter.

5.2. Pour Isopropanol and 70% Ethanol into weigh boats immediately prior to use.

5.3. Ensure that ALINE beads have reached room temperature prior to starting 2X bead clean up of excess 3' adapters.

5.4. NaOAc, ALINE beads and isopropanol must be added stepwise, and not premixed together prior to addition. Premixing results in a significant reduction in yield.

5.5. Ensure that sample is mixed 10X after each reagent (NaOAc, Bead, and IPA) addition.

5.6. Specific volumes are highlighted below. Note that two bead cleans are performed post 3' ligation. Note that DEPC-treated water elution volumes are different for the first and second bead clean.

5.7. Thoroughly mix the beads before the second bead clean up.

Bead clean #1

DNA volume (µL)	3M NaOAc (µL)	Bead Volume (µL)	IPA Volume (µL)	Bead Binding Time (mins)	Magnet Clearing Time (mins)	Supernatant Volume (µL)	2x 70% EtOH Wash Vol (µL)	Ethanol Air Dry Time (mins)	DEPC Elution Volume (µL)	Elution Time (mins)	Magnet Elution Time (mins)	Transfer Volume (µL)
20	25	40	60	15	7	153	150	5	22	3	2	20

Bead clean #2

DNA volume (µL)	3M NaOAc (µL)	Bead Volume (µL)	IPA Volume (µL)	Bead Binding Time* (mins)	Magnet Clearing Time (mins)	Supernatant Volume (µL)	2x 70% EtOH Wash Vol (µL)	Ethanol Air Dry Time (mins)	DEPC Elution Volume (µL)	Elution Time (mins)	Magnet Elution Time (mins)	Transfer Volume (µL)
20	25	40	60	15	7	153	150	5	10	3	2	9

5.8. Proceed immediately to 5' adapter ligation or alternatively, store 3'Ligated template overnight at -80°C.

6. 5' Adapter Ligation

6.1. Retrieve 1uM 5'miRNA adapter from -80°C and thaw at room temperature and then immediately return to ice.

6.2. Dispense adapter into one well of a 96-well plate, cover with VWR foil and then quick spin the plate. Denature the 5' adapter by incubating at 70°C for 2 minutes in a tetrad. Immediately snap chill the adapter for 1 minute. Quick spin plate and return to ice.

6.3. Transfer 2uL of 1uM 5' Adapter into empty wells of an AB1000 plate. Use a multichannel pipette to transfer all of the template to the adapter and mix 10X. Return the plate to ice.

6.4. Prepare 5' ligation brew. The volume requirement for 1 reaction set up is as follows:

Solution	1 rxn (µL)
3'Ligated template + denatured 5' adapter	11
10X T4 RNA ligase buffer	2
10 mM ATP, molecular grade	2
50% PEG 8000	3.5
Rnase Out	0.5
Ambion T4 RNA ligase	1
**Reaction volume**	**20**

miRNA_5'LigBrew (9µL)

6.5. Generate the 5'miRNALig Brew Mix calculator using LIMS:

6.6. Obtain the 1D large Solution/Box/Kit Label and Chemistry Label. Prepare the brew in an appropriate sized tube according to the chemistry calculator.

LIMS: Mix Standard Solutions > **miRNA_5'LigBrew**

6.7. Prepare brew according to the LIMS calculator. Prepare enough reactions to provide dead volume for brew preparation and dispense.

6.8. While on ice, carefully dispense 9 uL of 5' ligation brew into empty wells using a single channel pipette. Use a multichannel pipette set at 16uL to transfer the entire template including adapter to the ligation brew and then mix 10X. Ensure that no liquid is retained in the tips. Cover plate with VWR foil and then quick spin plate. Double check that volumes are even prior to starting the incubation.

6.9. Incubate plate for one hour at 37°C using the following tetrad protocol.

MIRNA>37LIG

6.10. Proceed immediately to first strand synthesis or alternatively, store samples at -80°C overnight.

7. First Strand Synthesis

7.1. Retrieve 20 uM RT primer from -20C and thaw at room temperature, and then immediately store on ice.

7.2. Dispense 2uL of RT primer into empty wells of an AB1000 plate. Use a multichannel pipette set at 16uL transfer the entire adapter ligated template to the RT primer and then mix 10X.

7.3. Cover plate with VWR foil and quick spin plate.

7.4. Incubate RT primer and ligated template at 65°C for 10 minutes on a tetrad. Set a timer. Snap chill plate on ice immediately after incubation. Incubate on ice for at least one minute and then quick spin and return to ice.

7.5. During the 10 minute incubation prepare RT brew. The volume requirement for 1 reaction set up is as follows:

Solution	1 rxn (µL)
Denatured Template + RT primer	22
5X First Strand buffer (contains DTT)	8
10mM dNTPs	2
Nuclease free water	6
Rnase Out	1
Maxima RT (200U/uL)	1
**Reaction volume**	**40**

miRNA_FirstStrand (18µL)

7.6. Generate the first strand brew mix calculator using LIMS:

LIMS: Mix Standard Solutions > **miRNA_FirstStrand**

7.7. Obtain the 1D large Solution/Box/Kit Label and Chemistry Label. Prepare the brew in an appropriate sized tube according to the chemistry calculator.

7.8. While on ice, transfer 18 uL of first strand brew to empty wells of an AB1000 plate. Use a multichannel pipette set at 30uL to transfer the denatured template plus primer to the first strand brew and mix 10X. Cover plate with VWR foil and then quick spin plate. Double check that volumes are even prior to starting the incubation.

7.9. Incubate plate for one hour at 44°C using the following tetrad protocol.

MIRNA>44Max

7.10. Proceed immediately to first strand purification or alternatively store the first strand template at -20°C.

8. First Strand Purification (Upper/Lower cut)

8.1. This step is essential to remove RT primer and reduce non-target products prior to PCR enrichment.

8.2. Prepare a P450 plate for dispensing NaOAc, ALINE beads, and DEPC-treated water. Pour 70% Ethanol and 100% Isopropanol into weigh boats just prior to use as described previously.

8.3. Ensure that ALINE beads have reached room temperature prior to starting 2X bead clean up excess 3' adapter.

8.4. Bead purification has been optimized to remove excess unwanted large and small products.

8.5. Ensure that sample is mixed 10X after each reagent (NaOAc, Bead, and IPA) addition.

8.6. Specific volumes are highlighted below. Note that the first bead clean step removes excess unwanted large products and supernatant is retained. NaOAc, ALINE Beads and IPA are then added to supernatant to capture the size of interest and the smallest products are washed away.

8.7. Thoroughly mix the beads before the lower cut size selection.

Upper Cut

DNA volume (µL)	Bead Volume (µL)	IPA Volume (µL)	Bead Binding Time (mins)	Magnet Clearing Time (mins)	Supernatant Transfer Volume (µL)
40	20	22	15	7	75

Lower Cut

Supernatant volume (µL)	3M NaOAc (µL)	Bead Volume (µL)	IPA Volume (µL)	Bead Binding Time* (mins)	Magnet Clearing Time (mins)	Supernatant Volume (µL)	2x 70% EtOH Wash Vol (µL)	Ethanol Air Dry Time (mins)	DEPC Elution Volume (µL)	Elution Time (mins)	Magnet Elution Time (mins)	Transfer Volume (µL)
75	10	20	68	15	7	173	150	5	20	3	2	20

8.8. Proceed immediately to PCR enrichment, or alternatively store the first strand template at -20°C for up to one week.

9. Indexed PCR enrichment and QC confirmation

9.1. Thaw the PE PCR primer 1.0 and miRNA indexing primer plate at a designated Pre-PCR work bench and then immediately place on ice. Quick spin the indexing primer plate prior to taking an aliquot.

9.2. To keep track of freeze-thaw cycles, mark off the indexing primer plate each time the plate is thawed even if it is not used.

9.3. The maximum freeze-thaw cycles for the indexing primer plate are 5 times.

9.4. iPCR brew (minus the primers) must be in a designated Pre-PCR laminar flowhood. Add PE PCR primer 1.0 to the brew at a designated pre-PCR work bench.

9.5. The volume requirement for 1 reaction set up is as follows:

Solution	1 rxn (µL)	
First Strand Template	19	
Ultrapure water	12.75	miRNA_iPCR (28.5µL)
5X Phusion HF buffer	10	
10 mM dNTPs	1.25	
DMSO	1.5	
Phusion hot start	0.5	
PE 1.0 primer (25uM)	2.5	
Index Primer (25uM)	2.5	
**Reaction volume**	**50**	

9.6. Generate the PCR Brew Mix calculator using LIMS.

9.7. Obtain the 1D large Solution/Box/Kit Label and Chemistry Label. Prepare the brew in an appropriate sized tube according to the chemistry calculator.

9.8. Dispense 2.5 µL of Index primer into each well containing template.

9.9. Add 28.5 µL of PCR reaction brew into each well of the destination plate containing the index primers. Mix 10 times with a multichannel pipette, cover with VWR foil and then quick spin plate. Check volumes prior to starting PCR run.

9.10. Run PCR program specified in the table below. Use a rubber pad on top of the reaction plate.

PCR parameters for others:

- 98˚C 60 sec
- 98˚C 15 sec
- 65˚C 30 sec
- 72˚C 15 sec
- 72˚C 5min
- 4˚C ∞

(98˚C 15 sec, 65˚C 30 sec, 72˚C 15 sec: Total of 15 cycles)

9.11. Run enriched samples on Agilent DNA 1000 assay to confirm that miRNA is present in each sample prior to pooling and gel purification. A miRNA peak will run at ~159bp on the DNA 1000 chip (Figure 2).

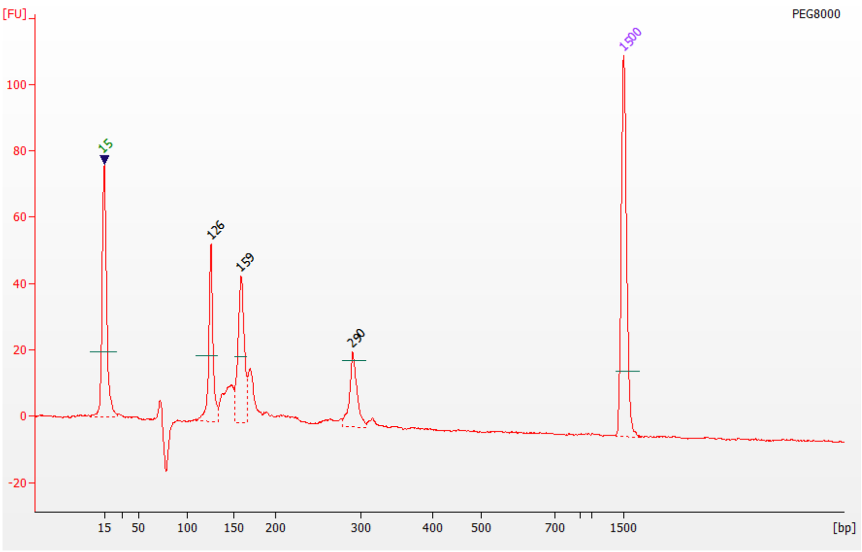

Figure 2. iPCR product prepared from 1 ug of human placental total RNA

9.12. After QC confirmation proceed immediately to PAGE or Barracuda size selection or alternatively store the indexed PCR product at -20C.

10. Gel Purification of miRNA libraries

10.1. Libraries are pooled according to the SOW of the collaborator. Your APC will provide the pooling strategy for a given project with the well location and volume required per well for size selection. The remaining template will be stored at -20C.

10.2. Prepare one 12% polyacrylamide gel per each pool you want to size select. Follow the recipe below for gel preparation. Make sure to mix the mixture well before addition of the last two components and then again after APS and TEMED addition.

12% POLYACRYLAMIDE GEL:

**REAGENT**	**Per GEL**
Ultrapure dH$_2$0	23.5 mL
40% Polyacrylamide (19:1 acrylamide:bis)	10.5 mL
50x TAE	700 µL
10% Ammonium Persulfate (APS)	350 µL
TEMED	30 µL

10.3. Let the gel polymerize for at least 40 minutes.

10.4. Set up the PAGE apparatus with cold water circulation and attach a label of colored tape to the gel plates, indicating the library name, date, and your initials.

10.5. Load 20µl of the 100bp DNA Ladder (20ng/µL) into one well and 25µL of 10bp DNA Ladder (20ng/µL) into an adjacent well near the center of the gel. Make sure there is no residual ladder on the outside of the tip when loading ladder into the well.

10.6. Add 10X loading dye to each of the pools, i.e 1uL dye/9uL template..

10.7. Split each pool over many wells – one pool per gel. On each gel load 20µL/well into wells on both sides of the ladders. Avoid the very edge wells if possible.

10.8. Immediately after loading, run the gel @ **200 V** for 6 hours. Change the running buffer half way through the run.

10.9. Using colored tape, attach a label to the gel apparatus which states the Pool name, start time, finish time, date, and your initials).

10.10. Dispose of all waste.

11. Gel Scan and Cutting DNA Fraction from PAGE

11.1. Put on a clean pair of gloves.

11.2. Set up sets of 0.5mL and 2mL tubes for shearing the gel slices: Make a hole through the bottom of 0.5mL tubes with an 18 gauge needle. Place each 0.5mL tube into a 2mL tube. You will one set of tubes per each pool.

11.3. Label each 2mL tube on the side with the pool, name, size fraction, date, and initials.

11.4. Cover the Dark Reader screen with a fresh sheet of plastic wrap.

11.5. Prepare fresh stain: 10µL stock in 100mL 1x TAE. Minimize exposure to light.

11.6. Stop the gel run after 6 hours and dismantle the PAGE apparatus.

11.7. Using a clean post PCR dedicated tray, stain the gel for at least 3 minutes.

.

11.8. Check the staining on the dark reader (ladders should be clearly visible). If it is stained well, retrieve the gel from the staining solution and place onto the Mylar sheet.

11.9. Log onto the computer and scan image on high sensitivity setting. Store the image in the appropriate network directory and file folder.

Name the file with Library Name_size selection__DateInitials,

i.e.MX050_ size selection gel_150606.gel

11.10. Print the image and paste it into your lab notebook.

11.11. Lay the Mylar sheet with the gel on top onto the Dark Reader and cut out the region containing miRNA which is around 155bp.

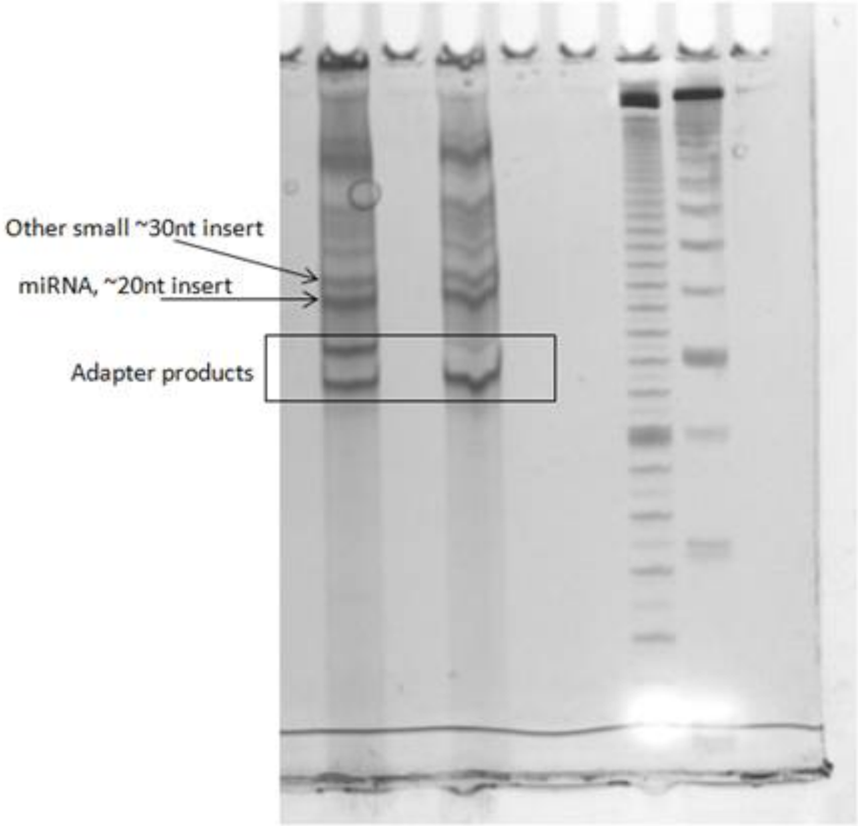

Figure 3.Polyacrylamide gel electrophoresis of a small RNA library pool.

11.12. Each well should look as above. Combine gel slices of each pool into separate 0.5mL tube prepared and labeled earlier for shearing.

11.13. Discard stain, spray with water, and wipe down tray. Discard Mylar sheet and remainder of the gel. Discard used blade in sharps container. Tidy up area. Change gloves.

11.14. Spin tubes at 12,000 rpm @ RT for 3 minutes. The gel slices should shear through the holes and collect into the bottom of the 2mL tubes.

11.15. After shearing check that all of the gel has cleared the 0.5 ml tubes. If no gel remains, discard the 0.5 mL tubes and add 500 µL of Elution buffer (5:1, LoTE:7.5M Ammonium Acetate) to each gel slurry. Mix well by vortexing. Pulse–spin.

11.16. If time permits, incubate for 1 hour at 65°C to elute DNA. If there is insufficient time to continue, incubate overnight at 4°C.

11.17. Clean PAGE apparatus: Run tap water over PAGE apparatus for 2 minutes; wipe down with 2% micro90; run water over PAGE apparatus for another 2 minutes. Wipe down the PAGE workstation.

12. Precipitate and Purify miRNA products

12.1. Put on a clean pair of gloves.

12.2. Retrieve the gel slurries from the previous day's PAGE gel from 4°C.

12.3. Vortex and pulse spin.

12.4. Heat the gel slurries at 65°C for 15 minutes in the preheated heat block.

12.5. Vortex the tubes, pulse-spin and transfer the gel slurry from each tube onto the top of a Spin-X filter column. Spin the sample through the spin column into the collection tube at 12,000 rpm for 3 minutes at 4°C.

12.6. Check each Spin Column tube and ensure that the entire buffer has spun through the filter. Re-spin the tubes if there is still liquid trapped in the gel material.

12.7. The total volume of eluate will depend on the amount of excised gel and the volume of added elution buffer; adjust the amount of ethanol used to precipitate the PCR products accordingly, and add the reagents to the eluate:

REAGENT	VOLUME
Eluate	500 µL
3 M Sodium Acetate	50 µL
Mussel Glycogen (20 mg/mL)	3 µL
100 % Ethanol	1380 µL
**TOTAL VOLUME**	**1933µL**

12.8. Vortex and pulse spin. Chill the tubes at -20°C for a minimum of 30 minutes.

12.9. Spin at 14,000 rpm / 4°C for 30 minutes.

12.10. Wash the pellet with 1 mL of 70% EtOH by adding the EtOH solution and inverting the tube. Make sure 70% EtOH is freshly made. Spin at 14,000 rpm / 4°C for 2 minutes. Discard the supernatant as previously.

12.11. Repeat the 70% EtOH wash.

12.12. Pulse spin the sample tube and carefully remove any residual ethanol by using a P200 pipette tip first to remove the majority of the supernatant, then finally using a P10 pipette tip to remove the last trace of solution. Mark the outside bottom of the tube to better locate the pellet when resuspending in buffer.

12.13. Allow the tube to air-dry for approximately 5 to 10 minutes at room temperature, until the white precipitate becomes translucent and is no longer visible.

12.14. Resuspend each sample in a total volume of 12 µL Qiagen EB buffer.

13. QC final product

13.1. Run the Agilent DNA 1000 Series II assay of size selected product by following LIBPR.0017.

13.2. Check the Agilent profile for the correct size fraction. The expected product of 155bp runs on Agilent Bioanalyzer at ~159bp on DNA 1000 (Figure 4). Discuss your results with your APC.

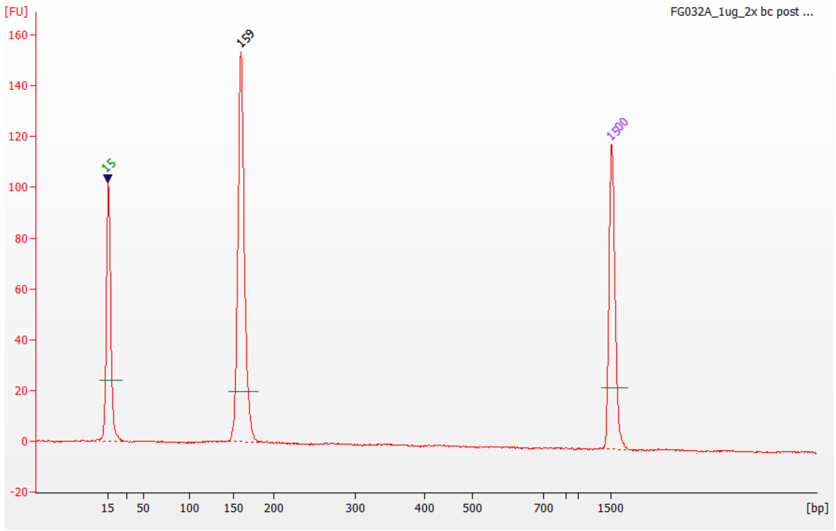

Figure 4. Final QC of PAGE-purified pooled miRNA libraries.

13.3. If samples are ready for submission, quantify 1µL of each sample with Qubit as per LIBPR.0030.
